# *Cryptococcus neoformans* Mar1 function links mitochondrial metabolism, oxidative stress, and antifungal tolerance

**DOI:** 10.3389/fphys.2023.1150272

**Published:** 2023-03-09

**Authors:** Calla L. Telzrow, Shannon Esher Righi, Jackson M. Cathey, Joshua A. Granek, J. Andrew Alspaugh

**Affiliations:** ^1^ Department of Medicine, Duke University School of Medicine, Durham, NC, United States; ^2^ Department of Molecular Genetics and Microbiology, Duke University School of Medicine, Durham, NC, United States; ^3^ Department of Microbiology and Immunology, Tulane University School of Medicine, New Orleans, LA, United States; ^4^ Department of Biostatistics and Bioinformatics, Duke University Medical Center, Durham, NC, United States

**Keywords:** fungi, mycoses, microbial pathogenesis, mitochondria, azole

## Abstract

**Introduction:** Microbial pathogens undergo significant physiological changes during interactions with the infected host, including alterations in metabolism and cell architecture. The *Cryptococcus neoformans* Mar1 protein is required for the proper ordering of the fungal cell wall in response to host-relevant stresses. However, the precise mechanism by which this *Cryptococcus*-specific protein regulates cell wall homeostasis was not defined.

**Methods:** Here, we use comparative transcriptomics, protein localization, and phenotypic analysis of a *mar1D* loss-of-function mutant strain to further define the role of *C. neoformans* Mar1 in stress response and antifungal resistance.

**Results:** We demonstrate that *C. neoformans* Mar1 is highly enriched in mitochondria. Furthermore, a *mar1*Δ mutant strain is impaired in growth in the presence of select electron transport chain inhibitors, has altered ATP homeostasis, and promotes proper mitochondrial morphogenesis. Pharmacological inhibition of complex IV of the electron transport chain in wild-type cells promotes similar cell wall changes as the *mar1*Δ mutant strain, supporting prior associations between mitochondrial function and cell wall homeostasis. Although Mar1 is not required for general susceptibility to the azole antifungals, the *mar1*Δ mutant strain displays increased tolerance to fluconazole that correlates with repressed mitochondrial metabolic activity.

**Discussion:** Together, these studies support an emerging model in which the metabolic activity of microbial cells directs cell physiological changes to allow persistence in the face of antimicrobial and host stress.

## Introduction

Mitochondria are the sites of many fundamental metabolic processes, including ATP generation from diverse carbon sources, lipid β-oxidation, amino acid synthesis, and reactive oxygen species (ROS) quenching. As a result, mitochondrial function is important for the viability and fitness of eukaryotic cells, both in permissive and stressful growth conditions. In the model yeast *Saccharomyces cerevisiae,* impairment in mitochondrial function, including respiration and lipid synthesis, causes structural defects in the fungal cell wall (Lussier et al., 1997; Pagé et al., 2003). Recent converging work in the human fungal pathogens *Candida* (She et al., 2015; She et al., 2016) and *Cryptococcus* (Caza et al., 2018; Horianopoulos et al., 2020) species has revealed that inhibition of mitochondrial function, either through genetic or chemical means, causes alterations in fungal cell wall structure and defects in virulence (Koch et al., 2019; Black et al., 2021). Given the extensive remodeling of the fungal cell wall that occurs in response to host-induced stressors, mitochondria-mediated cell wall processes can have major implications in fungal virulence. The mechanisms by which mitochondria contribute to adaptive cell wall remodeling processes remain largely unclear.

Mitochondrial function also contributes to fungal cell viability and growth in the presence of antifungal drugs. Several studies have demonstrated that alterations in mitochondrial function can affect resistance to echinocandins and azoles in *S. cerevisiae*, *Cryptococcus neoformans*, and *Candida* species (Chamilos et al., 2006; Šarinová et al., 2007; Dagley et al., 2011; Sun et al., 2013). Fungal pathogens can also develop antifungal tolerance, or the ability of a subpopulation of drug-susceptible cells to survive and grow slowly in the presence of the drug (Rosenberg et al., 2018; Berman and Krysan, 2020). The mechanisms underlying antifungal tolerance are being actively explored, and early work suggests that tolerance can be driven by heterogeneity in metabolic activity within a population of fungal cells (Rosenberg et al., 2018; Berman and Krysan, 2020). In contrast, more detailed mechanisms of antimicrobial tolerance among bacterial species have been characterized and may be relevant for fungi. For example, respiratory burst-generated ROS can inhibit enzymes involved in the *Staphylococcus aureus* tricarboxylic acid (TCA) cycle, leading to altered bacterial metabolic activity and increased tolerance to multiple antibiotics (Rowe et al., 2020). These converging observations suggest that altered primary cellular metabolism contributes to drug tolerance in both eukaryotes and prokaryotes. Additionally, these observations also suggest that mitochondrial function, specifically its roles in metabolism, may specifically contribute to drug tolerance in eukaryotic pathogens.

Previous work from our laboratory group identified the Mar1 protein as a *Cryptococcus*-specific protein lacking identifiable domains except for two predicted hydrophobic transmembrane domains (Esher et al., 2018). Phenotypic characterization of the *mar1*Δ loss-of-function mutant strain determined that Mar1 is required for cell wall remodeling, as the *mar1*Δ mutant strain possesses an intrinsically disorganized cell wall that is further disordered during incubation in host-like conditions. Stress-induced cellular changes in the *mar1*Δ mutant strain include increased exposure of immunogenic cell wall carbohydrates such as chitin, due to decreased levels of glucans and mannans in the outer cell wall layers, as well as reduced attachment of the capsular polysaccharides. Together, these cell surface changes result in increased activation of innate immune cells such as macrophages, likely mediated by the pattern recognition receptors TLR-2 and dectin-1 (Esher et al., 2018). These cell wall remodeling defects have important implications in pathogenesis. The *mar1*Δ mutant strain is hypovirulent in multiple models of *C. neoformans* infection (Esher et al., 2018). In contrast to animals inoculated with wild-type (WT) *C. neoformans* strains that die within 3 weeks of infection, many mice inoculated with the *mar1*Δ mutant strain survive for several months after infection (Esher et al., 2018; Telzrow et al., 2022). Moreover, the *mar1*Δ mutant strain-inoculated mice form fungi-containing pulmonary granulomas, or foci of chronic inflammation, in which *C. neoformans* cells persist without causing the death of the infected host (Telzrow et al., 2022).

We previously hypothesized that Mar1 may contribute to host-induced cell wall remodeling by serving as a component of intracellular trafficking machinery, as the *mar1*Δ mutant strain has defective translocation of the β-1,3-glucan synthase Fks1 from the cytosol to the cell surface in host-like conditions (Esher et al., 2018). In this work, we report that Mar1 is not required for canonical endoplasmic reticulum (ER)-Golgi trafficking. Instead, we find that Mar1 likely contributes to cell wall remodeling by regulating mitochondrial function. Mar1 localizes to the mitochondria and is required for normal mitochondrial metabolic activity. Specifically, Mar1 supports proper electron transport chain function and maintains mitochondrial homeostasis, including mitochondrial mass and mitochondrial membrane potential, particularly in the presence of host-like stress. Furthermore, we find that short-term inhibition of WT strain mitochondrial function in the presence of host-like stress is sufficient to induce *mar1*Δ mutant strain-like cell wall defects.

The *mar1*Δ mutant strain also displays other significant mitochondria-related phenotypes such as enhanced susceptibility to oxidative stress and increased fluconazole tolerance. Additionally, we find that fluconazole-tolerant *mar1*Δ mutant strain cells have reduced mitochondrial mass and mitochondrial membrane potential compared to non-tolerant cells. Together, these observations suggest that suppressed mitochondrial metabolism contributes to antifungal tolerance, complementing similar observations made in bacteria regarding antibacterial tolerance (Rowe et al., 2020). Mitochondrial metabolism has also recently been implicated in *C. neoformans* persistence both *in vitro* and *in vivo* (Alanio et al., 2015; Hommel et al., 2019), suggesting that altered fungal mitochondrial metabolic activity is a general mechanism of stress adaptation, promoting tolerance to antimicrobials as well as to the infected host (Alanio, 2020).

## Materials and methods

### Strains, media, and growth conditions

All *C. neoformans* strains used in this study were generated in the *C. neoformans* var. *grubii* H99 (*MAT*α) (Perfect et al., 1980), KN99 (*MAT*α), and KN99 (*MAT*a) (Nielsen et al., 2003) backgrounds. Strain details can be found in Table 1. All strains were recovered from glycerol stocks and were routinely maintained on yeast extract-peptone-dextrose (YPD) medium (1% yeast extract, 2% peptone, 2% dextrose, and 2% agar for solid medium). Unless otherwise indicated, strains were incubated at 30°C.

**TABLE 1:** Strains used in this study. All strains used in this study are included, along with the genotype and source of each strain.

Strain	Genotype	Source
H99	*MAT*⍺	Perfect et al. (1980)
MAK1	*MAT*⍺ *mar1*∆::*NAT*	Esher et al. (2018)
MAK11	*MAT*⍺ *mar1*∆::*NAT* + *MAR1-NEO*	Esher et al. (2018)
SKE106	*MAT*⍺ *mar1*∆::*NAT* + *H-GFP-MAR1-NEO*	Esher et al. (2018)
CUX48	*MAT*⍺ *kcs1*∆::*NEO*	Lev et al. (2015)
CUX98	*MAT*⍺ *kcs1*∆::NEO + *KCS1-NAT*	Lev et al. (2015)
CLT73	*MAT*⍺ *glo3*∆::*NAT*	Madhani, 2016
CLT85	*MAT*⍺ *glo3*∆::*NAT* + *H-mCherry-GLO3-NEO*	This study
CLT81	*MAT*⍺ *mar1*∆::*NAT* + *glo3*∆::*NAT*	This study
CLT82	*MAT*a *mar1*∆::*NAT* + *glo3*∆::*NAT*	This study
CLT83	*MAT*⍺ *mar1*∆::*NAT* + *glo3*∆::*NAT*	This study
TOC35	*MAT*⍺ *rim101*∆::*NAT*	O’Meara et al. (2010)
yAS40	*MAT*⍺ *SOD1-FLAG-HYG* + *SOD2-HA-NEO*	This study
CLT151	*MAT*⍺ *SOD1-FLAG-HYG* + *SOD2-HA-NEO* + *mar1*∆::*NAT*	This study

For permissive growth conditions, strains were incubated in liquid YPD medium at 30°C with 150 rpm shaking (Esher et al., 2018). For host-like growth conditions, strains were incubated in liquid CO_2_-independent tissue culture medium (Gibco) or RPMI 1640 medium (Sigma-Aldrich) (tissue culture [TC] medium) at 37°C with 150 rpm shaking (Esher et al., 2018).

### RNA sequencing preparation and analysis

The WT strain and the *mar1*Δ mutant strain were incubated in YPD medium at 30°C for 18 h. Approximately 10^9^ cells from each strain were pelleted, resuspended in YPD medium at 30°C and TC medium at 37°C, and subsequently incubated for 90 min. This experiment was conducted with six biological replicates for the WT strain and the *mar1*Δ mutant strain under both conditions (24 samples total). All samples were pelleted, flash frozen on dry ice, and lyophilized overnight. RNA was prepared and sequenced as previously described with some modifications (Brown et al., 2020). RNA was isolated using the Qiagen RNeasy Plant minikit with on-column DNase digestion (Qiagen, Valencia, CA). RNA quantity and quality were assessed using the Agilent 2,100 Bioanalyzer. The Ribo-Zero rRNA Removal Kit (Yeast) (Illumina, San Diego, CA, USA) was used for selective depletion of rRNA and enrichment of mRNA and ncRNA, and the NEBNext Ultra^TM^ II directional RNA library prep kit for Illumina was used to prepare sequencing libraries (New England Biolabs, Ipswich, MA). Libraries were submitted to the Duke University Sequencing and Genomic Technologies Shared Resource for sequencing on the Illumina NextSeq 500 with 75 base pair, single-end reads.

Reads were mapped to the *C. neoformans* H99 reference genome (obtained from NCBI, accessed March 2020) using STAR alignment software (Dobin et al., 2013). As part of data validation, Integrative Genomics Viewer (IGV) was used to visualize reads mapping to the *MAR1* locus. As expected, we observed a drastic reduction in reads mapping to the *MAR1* locus in the *mar1*Δ mutant strain samples. However, we made two observations about *MAR1* expression in this experiment. First, we noted expression from the 3′ end of the *MAR1* gene that included the last exon and the 3′ UTR in the *mar1*Δ mutant strain samples (Supplementary Figure S1A). The 3′ end of the *MAR1* locus overlaps with the 3′ end of CNAG_06694; when we previously constructed the *mar1*Δ mutant strain, we intentionally designed the *MAR1* knock-out construct to avoid affecting the expression, translation, or function of CNAG_06694, which likely explains the 3′ remnant of *MAR1* in the *mar1*Δ mutant strain samples. The fact that the majority of the *MAR1* domain of unknown function is not transcribed in the *mar1*Δ mutant strain, as well as the fact that every tested phenotype has been rescued by complementation with the full *MAR1* gene, suggests that this expressed 3’ remnant is nonfunctional. Additionally, we noted that one *mar1*Δ mutant strain sample incubated in YPD medium at 30°C displayed reads mapping throughout the complete *MAR1* ORF (Supplementary Figure S1A). Upon principal component analysis, this same sample clustered more closely with WT strain samples incubated in TC medium at 37°C than other *mar1*Δ mutant strain samples incubated in YPD medium at 30°C, suggested that this sample was mislabeled or contaminated (Supplementary Figure S1B). As a result, we dropped this sample from further analysis (Supplementary Figure S1B).

Differential expression analyses were performed in R using a Bioconductor workflow followed by the DESeq2 package with a false discovery rate (FDR) of 5% (Love et al., 2015). Two datasets were generated through this process. For the purposes of this study, we only included analyses of the WT strain and *mar1*Δ mutant strain samples incubated in TC medium at 37°C. Genes were considered statistically differentially expressed if they had adjusted *p* values <0.05. Significantly differentially expressed genes with log_2_ fold changes of at least ±1 were prioritized for downstream analyses.

FungiFun v2.2.8 was used to perform FunCat enrichment analyses to identify significantly overrepresented molecular functions (Priebe et al., 2011). Analysis was performed using default settings, including a 5% false discovery rate with a Benjamini-Hochberg correction.

### Mitochondrial fractionation

Subcellular fractionations were performed to isolate crude mitochondrial fractions and cytosolic fractions as previously described with a few modifications (Smith et al., 2021). The WT strain, the GFP-Mar1 strain, the Sod1-FLAG + Sod2-HA strain, and the Sod1-FLAG + Sod2-HA + *mar1*Δ strain were incubated in YPD medium at 30°C for 18 h. Cultures were harvested and resuspended in 2 mL/g wet weight cell pellet in DTT Buffer (100 mM Tris [pH 9.4], 10 mM DTT) and incubated at 30°C, 70 rpm for 20 min. To generate spheroplasts, cell pellets were resuspended in 6.5 mL/g wet weight cell pellet in lytic buffer (100 mM sodium citrate [pH 5.5], 1.1 M sorbitol) supplemented with 25 mg/mL lysing enzymes from *Trichoderma harzianum* (Sigma-Aldrich) and incubated at 30°C, 70 rpm for 3 h. Spheroplasts were pelleted at 2,200 x g for 8 min, gently washed in 7 mL/g wet weight cell pellet of ice-cold homogenization buffer (10 mM Tris [pH 7.4], 0.6 M sorbitol), and resuspended in homogenization buffer with 1X fungal specific protease inhibitor cocktail (Sigma-Aldrich). Spheroplasts were lysed by Dounce homogenization and diluted 1:1 with cold homogenization buffer with 1X fungal specific protease inhibitor cocktail. Cytosolic fractions (supernatants) were collected from crude mitochondrial fractions (pellets) by centrifugation. Protein contents were normalized by BCA assay, mixed with 4X Laemmli sample buffer (BioRad) under reducing conditions, and boiled for 10 min at 90°C. Normalized protein was loaded on a NuPage 4–12% Bis-Tris gel (Invitrogen) and western blots were performed as previously described (Esher et al., 2016). To detect Gapdh, blots were incubated with an anti-Gapdh polyclonal antibody (1/25,000 dilution; abcam ab9485; lot GR3386102-2) and an anti-rabbit peroxidase-conjugated secondary antibody (1/20,000 dilution; Jackson ImmunoResearch Laboratories, Inc. 115-035-174; lot 127837). To detect Cox1, blots were incubated with an anti-Cox1 monoclonal antibody (3 μg/mL dilution; abcam ab110270; lot GR3404565-1) and an anti-mouse peroxidase-conjugated secondary antibody (1/25,000 dilution; Jackson ImmunoResearch Laboratories, Inc. 111–035-008; lot 128022). To detect GFP-Mar1, blots were incubated with an anti-GFP monoclonal primary antibody (1/5,000 dilution; Roche Applied Science 11814460001; lot 14717400) and an anti-mouse peroxidase-conjugated secondary antibody (1/25,000 dilution; Jackson ImmunoResearch Laboratories, Inc. 111–035-008; lot 128022). To detect Sod1-FLAG, blots were incubated with an anti-FLAG monoclonal primary antibody (1/2,500 dilution; Sigma-Aldrich F3165; lot SLBQ7119V) and an anti-mouse peroxidase-conjugated secondary antibody (1/25,000 dilution; Jackson ImmunoResearch Laboratories, Inc. 111–035-008; lot 128022). To detect Sod2-HA, blots were incubated with an anti-HA monoclonal primary antibody (1/5,000 dilution; Roche Applied Science 11666606001; lot 15782900) and an anti-mouse peroxidase-conjugated secondary antibody (1/25,000 dilution; Jackson ImmunoResearch Laboratories, Inc. 111–035-008; lot 128022).

### Growth assays

To assess the impacts of Mar1 on growth in the presence of carbon deprivation, the WT strain, the *mar1*Δ mutant strain, and the *mar1*Δ + *MAR1* complemented strain were incubated in YPD medium at 30°C for 18 h. Cultures were washed once in 1X PBS and subsequently normalized by optical density at 600 nm (OD_600_) in 1X PBS. Serial dilutions were spotted onto standard YPD and yeast nitrogen base (YNB) medium agar plates with differing concentrations of glucose: 2%, 1%, 0.1%, 0.01%. Plates were incubated at 30°C and imaged daily.

To assess the impacts of Mar1 on growth in the presence of alternative carbon sources, the WT strain, the *mar1*Δ mutant strain, the *mar1*Δ + *MAR1* complemented strain, the *kcs1*Δ mutant strain, and the *kcs1*Δ + *KCS1* complemented strain were incubated in YPD medium at 30°C for 18 h. Cultures were washed once in 1X PBS and subsequently normalized by OD_600_ in 1X PBS. Serial dilutions were spotted onto standard YP (yeast extract-peptone) and YNB medium agar plates with differing carbon sources: glucose (2%), galactose (2%), glycerol (2%), acetate (2%), ethanol (2%), butyric acid (0.01%), ethanol + glucose (2% of each), and butyric acid (0.01%) + glucose (2%). Plates were incubated at 30°C and imaged daily.

To assess the impacts of Mar1 on growth in the presence electron transport chain inhibition, the WT strain, the *mar1*Δ mutant strain, and the *mar1*Δ + *MAR1* complemented strain were incubated in YPD medium at 30°C for 18 h. Cultures were washed once in 1X PBS and subsequently normalized by OD_600_ in 1X PBS. Serial dilutions were spotted onto standard YPD medium agar plates supplemented with differing electron transport chain inhibitors: rotenone (0.5 mg/mL) (Sigma-Aldrich), salicylhydroxamic acid (SHAM) (2.5 mM) (Sigma-Aldrich), carboxin (0.1 mg/mL) (Sigma-Aldrich), antimycin A (3 μg/mL) (Sigma-Aldrich), and sodium azide (NaN_3_) (0.5 mM) (Sigma-Aldrich). Rotenone and SHAM were reconstituted in DMSO. Carboxin was reconstituted in acetone. Antimycin A was reconstituted in 95% ethanol. NaN_3_ was reconstituted in water. Serial dilutions were also spotted onto standard YPD medium agar plates supplemented with equivalent volumes of each solvent. Plates were incubated at 30°C and imaged daily.

To compare the growth phenotypes of the *mar1*Δ mutant strain and the *glo3*Δ mutant strain, relevant strains were incubated in YPD medium at 30°C for 18 h. Cultures were washed once in 1X PBS and subsequently normalized by OD_600_ in 1X PBS. Serial dilutions were spotted onto standard YPD medium agar plates supplemented with different stressors: alkaline pH (pH 8), NaCl (1.5 M), SDS (0.03%), Congo red (0.5%), calcofluor white (1 mg/mL), and brefeldin A (50 μg/mL). Plates were incubated at 30°C, unless otherwise indicated, and imaged daily.

### Mitochondrial morphology analysis

The WT strain, the *mar1*Δ mutant strain, and the *mar1*Δ + *MAR1* complemented strain were incubated in YPD medium at 30°C and TC medium at 37°C for 18 h. Approximately 10^7^ cells from each sample were stained with 250 nM nonyl acridine orange (NAO) (Invitrogen) for 30 min at 30°C, 150 rpm shaking. All samples were then washed three times with 1X PBS and imaged by epifluorescence microscopy using a Zeiss Axio Imager A1 microscope equipped with an Axio‐Cam MRm digital camera. Cells were imaged by DIC and with a GFP filter to capture NAO staining. Identical exposure times were used to image all cells. Fiji software was used to process images. Mitochondrial morphology was assigned to cells using previously published criteria (Chang and Doering, 2018). Statistically significant differences in the percentages of cells within each strain with diffuse morphology were analyzed by one-way analysis of variance (ANOVA) and the Tukey-Kramer test (GraphPad Software, San Diego, CA).

### Mitochondrial mass and membrane potential quantification

The WT strain, the *mar1*Δ mutant strain, and the *mar1*Δ + *MAR1* complemented strain were incubated in YPD medium at 30°C and TC medium at 37°C for 18 h. Approximately 10^7^ cells from each sample were co-stained with 250 nM nonyl acridine orange (NAO) (Invitrogen) and 100 nM MitoTracker^TM^ Red CMXRos (MT) (Life Technologies) for 30 min at 30°C, 150 rpm shaking. Cells were concurrently stained with 10 μM Sytox^TM^ Blue (SB) (Invitrogen) for 15 min at 30°C, 150 rpm shaking. Unstained cells were prepared alongside of stained samples. All samples were then washed three times with 1X PBS. Approximately 10^6^ cells from each sample were submitted to the Duke Cancer Institute Flow Cytometry Shared Resource for analysis on a BD Fortessa X-20 flow cytometer. NAO was excited using a 488 nm laser, MT was excited using a 516 nm laser, and SB was excited using a 405 nm laser. Data were analyzed using FlowJo v10.8.0 software (FlowJo, LLC). Relevant events were gated for live cells (FSC vs. SSC and SSC vs. SB), single cells (FSC-H vs. FSC-A), and positive labelling (SSC vs. NAO/MT, determined using unstained cells as negative controls).

### ATP quantification analyses

The WT strain, the *mar1*Δ mutant strain, and the *mar1*Δ + *MAR1* complemented strain were incubated in YPD medium at 30°C and TC medium at 37°C for 18 h. Fungal cells were lysed and total lysates were collected as previously described (Esher et al., 2018). Briefly, cultures were pelleted, flash frozen on dry ice, and lysed by bead beating. Lysates were collected in 1.4 mL NP40 lysis buffer (6 mM Na_2_HPO_4_, 4 mM NaH_2_PO_4_, 1% Nonidet P-40, 150 mM NaCl, 2 mM EDTA, 1X protease inhibitors [Complete mini, EDTA-free; Roche Applied Science], 1X phosphatase inhibitors [Phos-Stop, Roche Applied Science], and 1 mM phenylmethylsulfonyl fluoride [PMSF]). Crude lysates were cleared by centrifugation at 2,500 x g at 4°C for 5 min. Total cell lysate protein concentrations were measured and normalized by BCA assay. Total cellular ATP was measured using a firefly luciferase bioluminescent assay (Invitrogen), following standard protocol. Statistically significant differences in total cellular ATP were analyzed by two-way analysis of variance (ANOVA) and the Tukey-Kramer test (GraphPad Software, San Diego, CA). Statistically significant differences in the percentage of ATP in TC medium at 37°C compared to YPD medium at 30°C were analyzed by one-way ANOVA and the Tukey-Kramer test (GraphPad Software, San Diego, CA).

### Cell wall chitin staining

Exposed cell wall chitin was stained and quantified as previously described with some modifications (Esher et al., 2018). The WT strain and the *mar1*Δ mutant strain were incubated in YPD medium at 30°C for 18 h. Cultures were diluted 1:10 in TC medium with and without 0.5 mM NaN_3_ and conditioned for 24 h at 37°C. Cells were washed twice in 1X PBS, fixed with 3.7% formaldehyde for 5 min at room temperature, and washed twice again with 1X PBS. Approximately 10^7^ cells from each sample were subsequently stained with 100 μg/mL FITC-conjugated wheat germ agglutinin (WGA, Molecular Probes) as previously described (Esher et al., 2018). Unstained cells were prepared alongside of stained samples. Approximately 10^6^ cells from each sample were submitted to the Duke Cancer Institute Flow Cytometry Shared Resource for analysis on a BD FACSCanto II flow cytometer. WGA was excited using a 488 nm laser. Data were analyzed using FlowJo v10.8.0 software (FlowJo, LLC). Relevant events were gated for live cells (FSC vs. SSC), single cells (FSC-H vs. FSC-A), and positive labelling (SSC vs. WGA, determined using unstained cells as negative controls). Representative images of samples were captured by epifluorescence microscopy using a Zeiss Axio Imager A1 microscope equipped with an Axio‐Cam MRm digital camera. WGA was imaged using a GFP filter. Identical exposure times were used to image all cells. Fiji software was used to process images. Statistically significant differences in the percentage of cells in each strain with exposed cell wall chitin were analyzed by two-way analysis of variance (ANOVA) and the Tukey-Kramer test (GraphPad Software, San Diego, CA).

To compare the cell wall chitin phenotypes of the *mar1*Δ mutant strain and the *glo3*Δ mutant strain, total cell wall chitin and exposed chitin were stained as previously described (Esher et al., 2018). The relevant strains were incubated in TC medium at 37°C for 18 h. Cells were washed twice in 1X PBS, fixed with 3.7% formaldehyde for 5 min at room temperature, and washed twice again with 1X PBS. Approximately 10^7^ cells from each sample were subsequently stained with 100 μg/mL FITC-conjugated wheat germ agglutinin (WGA, Molecular Probes) for 35 min in the dark, followed by 25 μg/mL calcofluor white (CFW) for 10 min (Esher et al., 2018). Unstained cells were prepared alongside of stained samples. Approximately 10^6^ cells from each sample were submitted to the Duke Cancer Institute Flow Cytometry Shared Resource for analysis on a BD FACSCanto II flow cytometer. WGA was excited using a 488 nm laser. CFW was excited using a using a 405 nm laser. Representative images of samples were captured by epifluorescence microscopy using a Zeiss Axio Imager A1 microscope equipped with an Axio‐Cam MRm digital camera. WGA was imaged using a GFP filter. CFW was imaged using a DAPI filter. Identical exposure times were used to image all cells. Fiji software was used to process images.

### Oxidative stress susceptibility assays

The WT strain, the *mar1*Δ mutant strain, and the *mar1*Δ + *MAR1* complemented strain were incubated in YPD medium at 30°C for 18 h. Cells were washed once in 1X PBS and subsequently normalized to an OD_600_ of 0.6 in 1X PBS. For each strain, 100 μL of cell solution were spread onto YPD medium agar plates. H_2_O_2_ (VWR Chemicals) and menadione (Sigma-Aldrich) susceptibilities were assessed by standard disc diffusion assays. For H_2_O_2_ susceptibility, 15 μL of differing concentrations of H_2_O_2_ were added to paper discs: 1.5%, 3%, 6% (% v/v). H_2_O_2_ was prepared in water, so a disk with an equivalent volume of water was included as a control. For menadione susceptibility, 5 μL of differing concentrations of menadione were added to paper discs: 2 mM, 10 mM, 50 mM. Menadione was reconstituted in 95% ethanol, so a disk with an equivalent volume of 95% ethanol was included as a control. Plates were incubated at 30°C and imaged daily. Zones of inhibition were measured as markers of antifungal susceptibility.

### Intracellular ROS staining

The WT strain and the *mar1*Δ mutant strain were incubated in YPD medium at 30°C for 18 h. For total intracellular ROS, approximately 10^7^ cells of each strain were transferred to fresh YPD medium and fresh YPD medium supplemented with 0.01% H_2_O_2_ (% v/v). Cells were stained with 10 μM 2′,7′-dichlorodihydrofluorescein diacetate (DCF) (Invitrogen) and subsequently incubated at 30°C, 150 rpm for 2 h. Unstained cells were prepared alongside of stained samples as controls. All samples were then washed three times with 1X PBS. Approximately 10^6^ cells from each sample were submitted to the Duke Cancer Institute Flow Cytometry Shared Resource for analysis on a BD Fortessa X-20 flow cytometer. DCF was excited using a 488 nm laser. Data were analyzed using FlowJo v10.8.0 software (FlowJo, LLC). Relevant events were gated for live cells (FSC vs. SSC), single cells (FSC-H vs. FSC-A), and positive labelling (SSC vs. DCF, determined using unstained cells as negative controls). Statistically significant differences in the percentage of total cells with measurable DCF staining were analyzed by two-way analysis of variance (ANOVA) and the Tukey-Kramer test (GraphPad Software, San Diego, CA).

For mitochondrial superoxide, approximately 10^7^ cells of each strain were transferred to fresh YPD medium, fresh YPD medium supplemented with 0.01% H_2_O_2_ (% v/v), and fresh YPD medium supplemented with 10 μg/mL fluconazole and subsequently incubated at 30°C, 150 rpm for 18 h. Cells were stained with 2.5 μM MitoSox Red (Invitrogen) for 30 min at 30°C. Unstained cells were prepared alongside of stained samples as controls. All samples were then washed three times with 1X PBS. Approximately 10^6^ cells from each sample were submitted to the Duke Cancer Institute Flow Cytometry Shared Resource for analysis on a BD Fortessa X-20 flow cytometer. MitoSox red was excited using a 516 nm laser. Data were analyzed using FlowJo v10.8.0 software (FlowJo, LLC). Relevant events were gated for live cells (FSC vs. SSC), single cells (FSC-H vs. FSC-A), and positive labelling (SSC vs. MitoSox Red, determined using unstained cells as negative controls). Statistically significant differences in the percentage of total cells with measurable MitoSox Red staining were analyzed by two-way analysis of variance (ANOVA) and the Tukey-Kramer test (GraphPad Software, San Diego, CA) or Student's *t* test.

### Fluconazole tolerance analyses

To explore the impacts of Mar1 on fluconazole tolerance, the WT strain, the *mar1*Δ mutant strain, and the *mar1*Δ + *MAR1* complemented strain were incubated in YPD medium at 30°C for 18 h. Cultures were normalized to an OD_600_ of 0.6, diluted 1:10 in 1X PBS, and 100 μL of cell solution was spread on YPD medium agar plates. An Etest strip (bioMérieux) containing a gradient of fluconazole concentrations was placed on top of each fungal lawn. Plates were incubated at 30°C for at least 96 h and imaged daily. Zones of inhibition were used to determine the minimum inhibitory concentration (MIC) for each tested fungal strain. Tolerant cells were hypothesized to be small colonies that appeared within the zone of inhibition by 96 h of incubation. To confirm that these colonies were a result of tolerance, single colonies within the zone of inhibition and single colonies outside of the zone of inhibition were isolated, resuspended in 1X PBS, and 100 μL of cell solution was passaged onto a fresh YPD medium agar plate with a new fluconazole Etest strip (Berman and Krysan, 2020). Again, plates were incubated at 30°C for at least 96 h and imaged daily. Tolerance was confirmed upon observance that colonies isolated from within the zone of inhibition reproduced the growth pattern of colonies isolated from outside of the zone of inhibition and the original *mar1*Δ mutant background strain (Berman and Krysan, 2020). The number of tolerant colonies appearing within each zone of inhibition was counted for each strain across multiple experiments. Statistically significant differences in the average number of colonies within the zone of inhibition for each strain were analyzed by one-way analysis of variance (ANOVA) and the Tukey-Kramer test (GraphPad Software, San Diego, CA).

## Results

### Mar1 is not a component of canonical ER-Golgi intracellular trafficking

We previously demonstrated that *C. neoformans* Mar1 is required for proper cell wall remodeling in response to *in vitro* host-like conditions (Esher et al., 2018), resulting in defective cell wall organization in the *mar1*Δ mutant strain and an enhanced TNF-α response by macrophages in fungal co-culture. Furthermore, we also reported that Mar1 is required for the proper intracellular trafficking of the β-1,3-glucan synthase Fks1 to the plasma membrane in response to this condition (Esher et al., 2018). Based on these data, we initially hypothesized that Mar1 may be a novel component of *C. neoformans* intracellular vesicular trafficking machinery. To test this hypothesis, we performed comparative phenotypic analyses between the *mar1*Δ mutant strain and the *glo3*Δ loss-of-function mutant strain, a strain lacking the ADP-ribosylation factor GTPase activating protein (Arf-GAP), Glo3, which is involved in ER-Golgi transport. The *glo3*Δ mutant strain did not share the *mar1*Δ mutant strain defects in growth at 37°C, nor hypersensitivity to the cell wall stressors calcofluor white or Congo red (Supplementary Figure S2A). Accordingly, the *glo3*Δ mutant strain did not demonstrate the same enhanced wheat germ agglutinin (WGA) staining pattern as the *mar1*Δ mutant strain in *vitro* host-like conditions (TC medium at 37°C), suggesting that the *glo3*Δ mutant strain does not share the stress-induced increase in exposed cell wall chitin associated with the *mar1*Δ mutation (Supplementary Figure S2B). Additionally, the *glo3*Δ mutant strain was predictably resistant to brefeldin A, an inhibitor of protein transport between the ER and Golgi complex (Supplementary Figure S2C). In contrast the *mar1*Δ mutant strain was fully susceptible to the activity of this compound, but not quite as susceptible as the *rim101*Δ mutant strain (O’Meara et al., 2010; Jung et al., 2016) (Supplementary Figure S2C). Collectively, these observations suggest that Mar1 is likely not a component of canonical Glo3-mediated ER-Golgi trafficking.

### Mar1 is required for the normal transcriptional response to host-like conditions, particularly for genes involved in mitochondrial function

**FIGURE 1:**
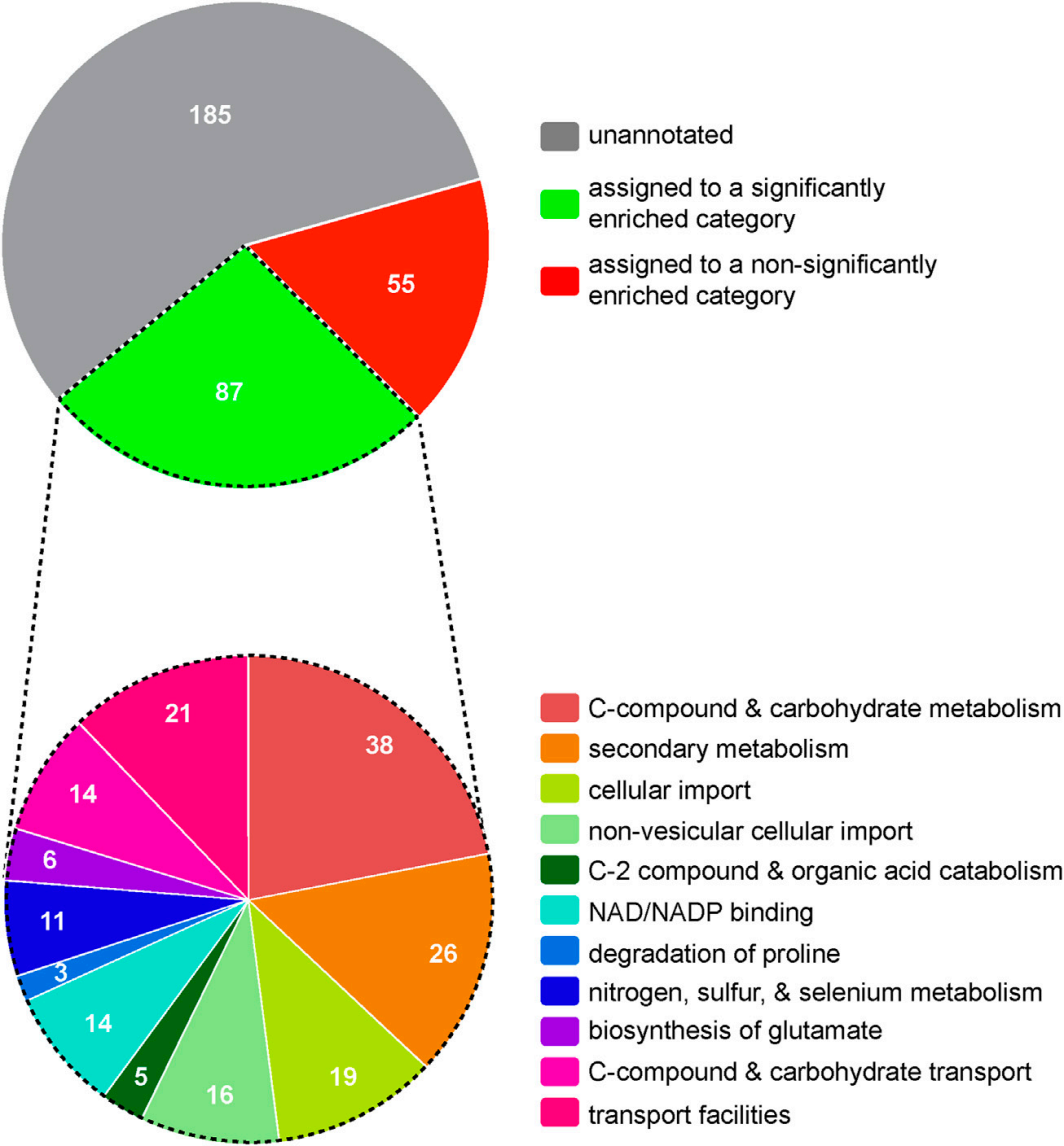
Hierarchical ontology classification enrichment analyses of RNA sequencing results. The WT strain and the *mar1*Δ mutant strain were conditioned in TC medium at 37°C and RNA sequencing analyses were subsequently performed. Prioritized significantly differentially expressed genes (log_2_ fold ±1 and adjusted *p*-values <0.05) were analyzed by FunCat enrichment analysis. All 327 prioritized significantly expressed genes are shown in the top pie chart, with only a subset (87 genes) being annotated and assigned to one or more significantly enriched functional categories. The functional category assignments of these 87 genes are shown in the bottom pie chart. Functional categories with an adjusted *p*-value <0.05, determined by Benjamini-Hochberg false discovery rate (FDR) correction, were considered significantly enriched. Pie charts are depicted to scale.

To explore potential mechanisms by which Mar1 contributes to cell wall remodeling, we next employed an RNA sequencing approach as an indirect measurement of cellular processes altered in the *mar1*Δ mutant strain. We conditioned the WT strain and the *mar1*Δ mutant strain in TC medium at 37°C, isolated rRNA-depleted RNA, and performed bulk RNA sequencing (Supplementary Table S1). Upon prioritizing genes with log_2_ fold changes of at least ±1 and adjusted *p*-values <0.05, we identified 327 genes that were differentially expressed in the *mar1*Δ mutant strain compared to the WT strain, specifically 145 induced genes and 182 repressed genes (Supplementary Tables S2, S3). Although the majority of these differentially expressed genes were unannotated with regard to predicted function, FunCat analysis (Priebe et al., 2011) assigned 87 of these genes to significantly enriched functional categories involved in metabolism and cellular transport (Figure 1; Supplementary Table S4). Many of these functional categories were involved in mitochondrial function, including “C-compound and carbohydrate metabolism”, “C-2 compound and organic acid catabolism”, “biosynthesis of glutamate”, “C-compound and carbohydrate transport”, and “transport facilities” (Figure 1, bottom; Supplementary Table S4). Furthermore, 22 of the differentially expressed genes assigned to these functional categories were predicted to localize to the mitochondria based on orthology and/or protein structure (Supplementary Table S4).

### Mar1 localizes to the mitochondria

**FIGURE 2:**
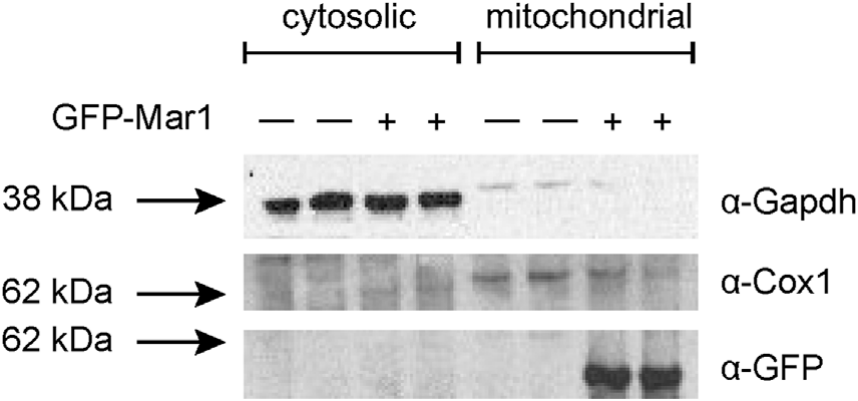
Mar1 mitochondrial localization analyses. Mitochondrial fractionations were performed on the WT strain and the GFP-Mar1 strain following incubation in YPD medium. Normalized cytosolic and crude mitochondrial lysates were separated by SDS-PAGE and western blots were performed probing for Gapdh with an anti-Gapdh antibody, Cox1 with an anti-Cox1 antibody, and GFP-Mar1 with an anti-GFP antibody. The numbers to the left of the blot represent molecular weight in kilodaltons (kDa). The approximate size of Gapdh is 35 kDa. The approximate size of Cox1 is 60 kDa. The approximate size of GFP-Mar1 is 60 kDa.

Based on the transcriptional dysregulation of genes involved in mitochondrial function in the *mar1*Δ mutant strain, we hypothesized that Mar1 may itself contribute to mitochondrial function. Previously published work with the GFP-Mar1 strain found that Mar1 localized to both cell surface-associated and cytosolic puncta. To determine whether this localization pattern included the mitochondria, we performed subcellular fractionations of the WT strain and the GFP-Mar1 strain to isolate crude mitochondrial fractions. By western blot analysis, we found GFP-Mar1 to be highly enriched in the crude mitochondrial fraction (confirmed by Cox1 enrichment) compared to the cytosolic fraction (confirmed by Gapdh enrichment) (Smith et al., 2021) (Figure 2). Computational analysis of the Mar1 protein sequence using MitoProt II (Claros and Vincens, 1996) further supported this experimental result, finding that Mar1 has a theoretical mitochondrial importability of 88%. Although Mar1 is nuclear-encoded and does not contain an N-terminal mitochondria-targeting presequence, measures of Mar1 sequence hydrophobicity, specifically the proximity between hydrophobic domains and the 17-residue region of higher hydrophobicity, likely drive the high probability of mitochondrial import for Mar1 (Claros, 1995; Claros and Vincens, 1996).

### Mar1 is required for normal electron transport chain function

**FIGURE 3:**
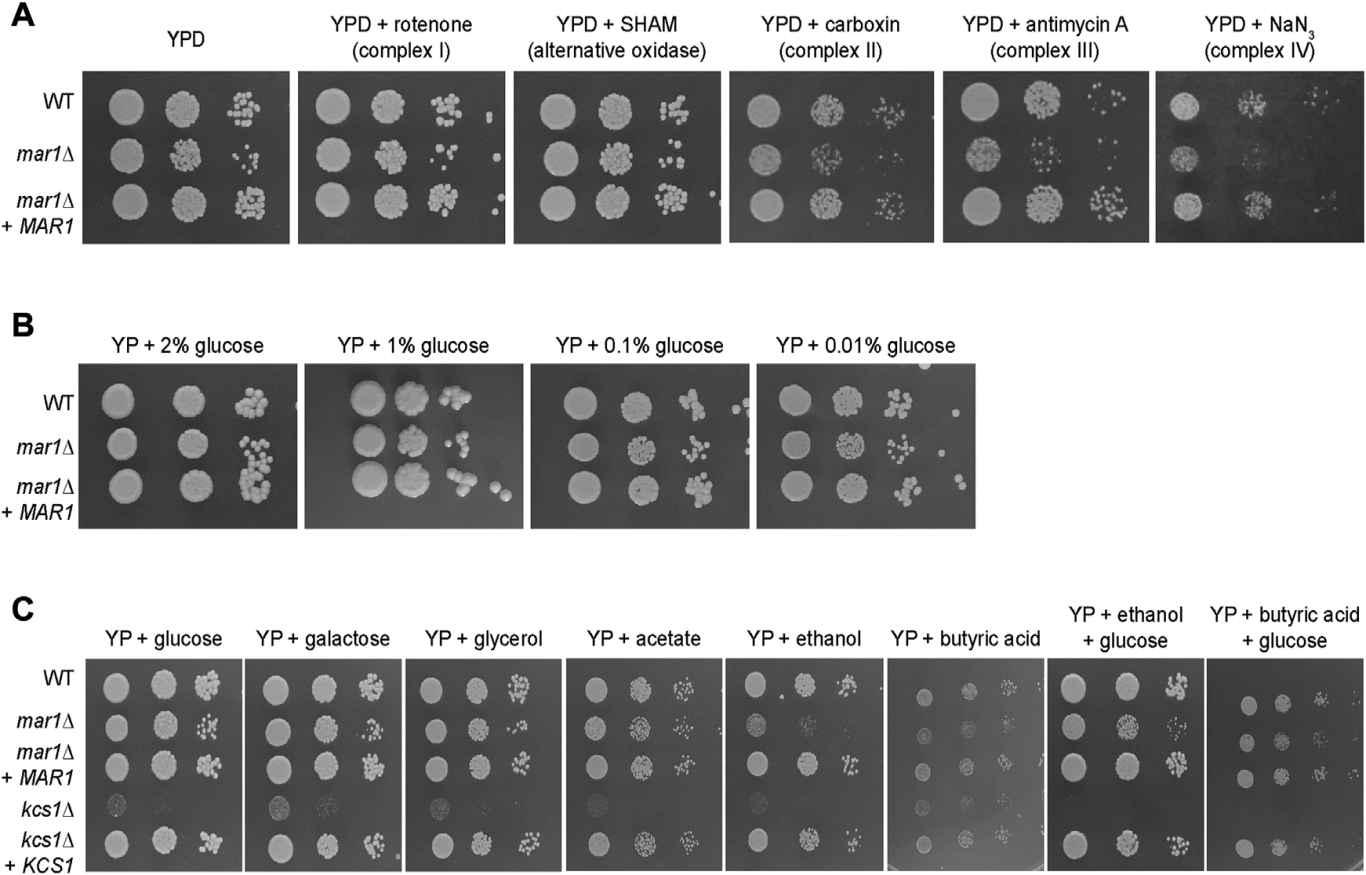
Mar1 contributions to electron transport chain function and carbon source utilization. **(A)** Growth phenotypes of the WT strain, the *mar1*Δ mutant strain, and the *mar1*Δ + *MAR1* complemented strain were assessed in the presence of electron transport chain inhibitors. Strains were normalized by OD_600_ and were subsequently grown on YPD medium agar plates supplemented with rotenone (0.5 mg/mL), salicylhydroxamic acid (SHAM) (2.5 mM), carboxin (0.1 mg/mL), antimycin A (3 μg/mL), and sodium azide (NaN_3_) (0.5 mM) at 30°C. Plates were imaged daily. Experiment was repeated two times to ensure reproducibility and representative images are shown. **(B)** Growth phenotypes of the WT strain, the *mar1*Δ mutant strain, and the *mar1*Δ + *MAR1* complemented strain were assessed in the presence of carbon deprivation. Strains were normalized by OD_600_ and were subsequently grown on YP medium agar plates supplemented with 2% glucose, 1% glucose, 0.1% glucose, and 0.01% glucose at 30°C. Plates were imaged daily. Experiment was repeated two times to ensure reproducibility and representative images are shown. **(C)** Growth phenotypes of the WT strain, the *mar1*Δ mutant strain, the *mar1*Δ + *MAR1* complemented strain, the *kcs1*Δ mutant strain, and the *ksc1*Δ + *KCS1* complemented strain were assessed in the presence of alternative carbon sources. Strains were normalized by OD_600_ and were subsequently grown on YP medium agar plates supplemented with glucose (2%), galactose (2%), glycerol (2%), acetate (2%), ethanol (2%), butyric acid (0.01%), ethanol + glucose (2% of each), and butyric acid (0.01%) + glucose (2%) at 30°C. Plates were imaged daily. Experiment was repeated two times to ensure reproducibility and representative images are shown.

To explore the role of Mar1 in mitochondrial processes such as oxidative phosphorylation and ATP synthesis, we assessed the ability of the *mar1*Δ mutant strain to grow on YPD medium supplemented with electron transport chain inhibitors. On YPD medium alone, the *mar1*Δ mutant strain demonstrated slightly impeded growth compared to the WT strain and the *mar1*Δ + *MAR1* complemented strain, as indicated by a smaller colony size on spotting assays, as previously demonstrated (Figure 3A; Supplementary Figure S3A) (Esher et al., 2018). Compared to YPD medium alone, the *mar1*Δ mutant strain displayed similar comparative growth to that of the WT strain and the *mar1*Δ + *MAR1* complemented strain in the presence of inhibitors of complex I (rotenone) and the alternative oxidase (salicylhydroxamic acid [SHAM]), with the *mar1*Δ mutant strain displaying a slight reduction in colony size (Figure 3A; Supplementary Figure S3A). However, the *mar1*Δ mutant strain displayed exacerbated growth attenuation in the presence of complex II (carboxin), complex III (antimycin A), and complex IV (sodium azide [NaN_3_]) inhibitors (Figure 3A; Supplementary Figure S3A). This growth attenuation was due to the activity of the inhibitors, and not the solvents used to solubilize them, as growth on YPD medium supplemented with these solvents mirrors growth on YPD medium alone (Supplementary Figure S3B).

Because our RNA sequencing analyses demonstrated that Mar1 is required for the normal transcriptional regulation of many genes involved in carbohydrate metabolism, we also explored the role of Mar1 in carbon source utilization. We observed that the *mar1*Δ mutant strain displayed similar growth to that of the WT strain and the *mar1*Δ + *MAR1* complemented strain in the presence of carbon deprivation (1%, 0.1%, and 0.01% glucose) in yeast-peptone (YP) medium (Figure 3B). Similar results were observed when growth was assessed on yeast nitrogen base (YNB) medium (Supplementary Figure S4A). Furthermore, we observed that the *mar1*Δ mutant strain displayed similar growth to that of the WT strain and the *mar1*Δ + *MAR1* complemented strain in the presence of alternative carbon sources (galactose, glycerol, and acetate) in YP medium (Figure 3C). These growth kinetics contrasted starkly with the documented growth defects of the *kcs1*Δ mutant strain, which is highly attenuated for growth on alternative carbon sources due to the role of Kcs1 as an inositol hexaphosphate kinase (Lev et al., 2015).

Although able to grow well on several carbon sources, the *mar1*Δ mutant strain displayed exacerbated growth attenuation when either ethanol or butyric acid was the sole available carbon source. Supplementation with glucose appeared to largely but incompletely rescue these phenotypes, suggesting that these phenotypes are, in part, likely due to the accumulation of toxic intermediates, and not simply due to an inability to utilize these carbon sources (Figure 3C). Similar results were observed when growth was assessed on YNB (Supplementary Figure S4B). Therefore, although Mar1 is dispensable for carbon source utilization, it is necessary for proper electron transport chain function.

### Mar1 is required for mitochondrial homeostasis in response to host-like stress

**FIGURE 4:**
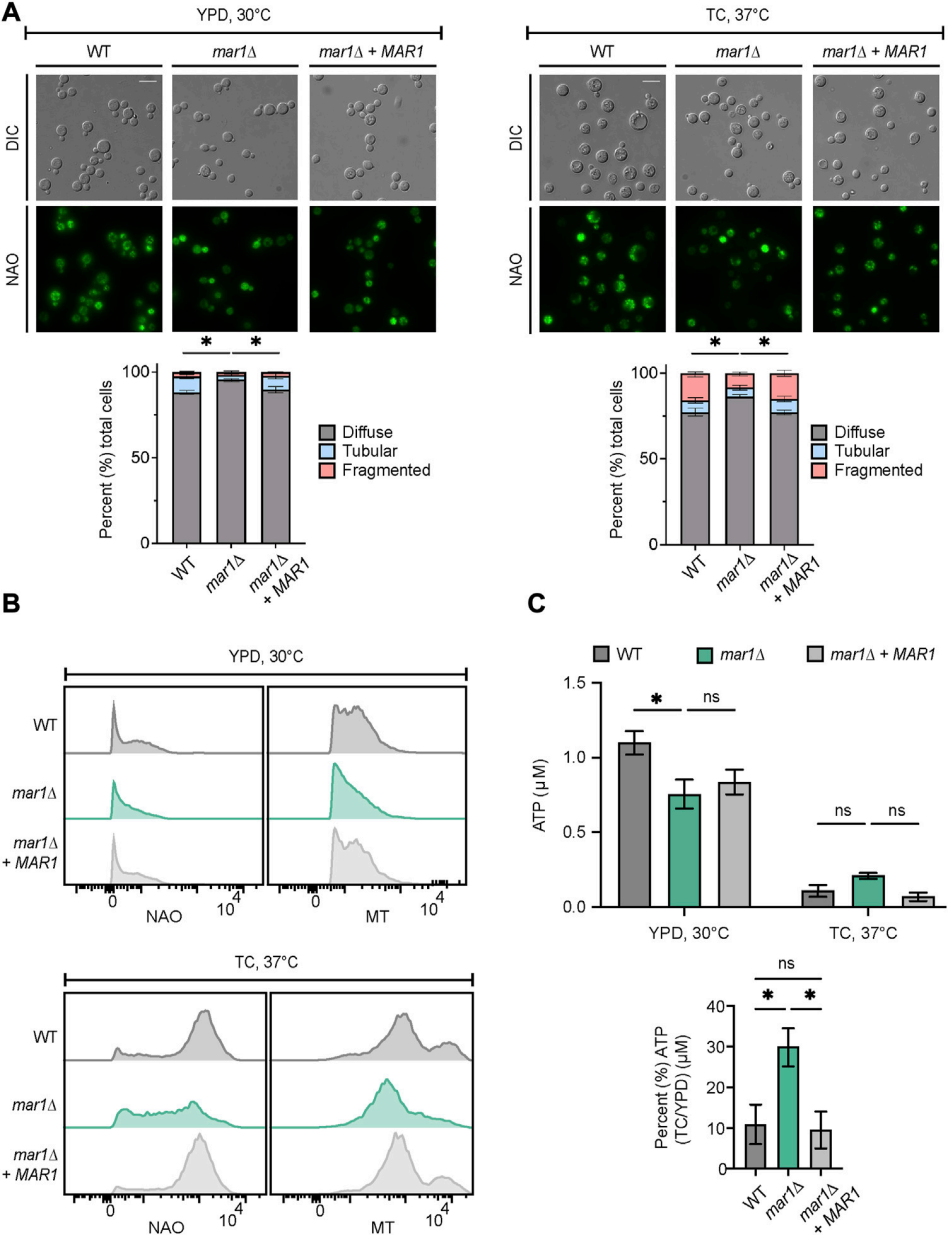
Mar1 contributions to mitochondrial homeostasis. **(A)** Mitochondrial morphologies of the WT strain, the *mar1*Δ mutant strain, and the *mar1*Δ + *MAR1* complemented strain were assessed following incubation in YPD medium at 30°C (left) and TC medium at 37°C (right). Mitochondria were stained with nonyl acridine orange (NAO) and subsequently imaged by epifluorescence microscopy (63x scale bar, 10 μm). Data are presented as the proportion of cells in each sample with diffuse, tubular, and fragmented morphologies. A minimum of 200 cells were analyzed across three biological replicates (*n* = 3). Error bars represent the SEM. Statistically significant differences in the percentage of cells in each strain with diffuse morphology were determined using a one-way ANOVA and the Tukey-Kramer test (*, *p* < 0.05). DIC, differential interference contrast. **(B)** Mitochondrial mass and mitochondrial membrane potential of the WT strain, the *mar1*Δ mutant strain, and the *mar1*Δ + *MAR1* complemented strain were measured following incubation in YPD medium at 30°C (top) and TC medium at 37°C (bottom). Mitochondria were co-stained with NAO, as a marker of mitochondrial mass, MitoTracker^TM^ Red CMXRos (MT), as a marker of mitochondrial membrane potential, and Sytox^TM^ Blue (SB). Flow cytometry was used to quantify staining intensity. Relevant events were gated for live cells (FSC vs. SSC and SSC vs. SB), single cells (FSC-H vs. FSC-A), and positive labelling (SSC vs. NAO/MT, determined using unstained cells as negative controls). Data are represented as histograms with mean fluorescence intensity on the x-axis and cell number of the y-axis. Experiment was repeated three times to ensure reproducibility. **(C)** Total cellular ATP was measured for the WT strain, the *mar1*Δ mutant strain, and the *mar1*Δ + *MAR1* complemented strain following incubation in YPD medium at 30°C and TC medium at 37°C. Normalized total cell lysates were analyzed for total cellular ATP using a firefly luciferase bioluminescent assay. Data are presented as the average ATP (μM) per sample (top) and the percentage of ATP in TC medium at 37°C compared to YPD medium at 30°C (μM) (bottom) across two biological replicates (*n* = 2). Error bars represent the SEM. Statistical significance was determined using two-way and one-way ANOVA and the Tukey-Kramer test (*, *p* < 0.05; ns, not significant).

Because the *mar1*Δ mutant strain displays its most drastic cell wall defects in the presence of host-like stress, we posited that Mar1 may be specifically required for mitochondrial adaptation to host-like stress, rather than directing metabolic shifts in response to different carbon sources. As an initial assessment of the mitochondrial stress response, we explored mitochondrial morphology by nonyl acridine orange (NAO) staining, a stain that binds mitochondrial cardiolipin, and as a result, stains mitochondria independent of their membrane potential. Consistent with previously reported observations, we observed three mitochondrial morphologies: diffuse, tubular, and fragmented (Chang and Doering, 2018). As previously described, in YPD medium incubated at 30°C, most cells in all strain backgrounds displayed a diffuse morphology (Figure 4A, left) (Chan, 2006; Chang and Doering, 2018). However, the *mar1*Δ mutant strain displayed a significantly larger proportion of cells with diffuse morphology (∼96%) compared to the WT strain (∼88%) and the *mar1*Δ + *MAR1* complemented strain (∼90%) (Figure 4A, left). In TC medium incubated at 37°C, all three strains displayed an increase in the percentage of cells with fragmented morphologies and a concordant decrease in cells with diffuse morphology (Figure 4A, right). These morphological changes were expected because the mitochondria often undergo fusion and fission in response to cellular stress, resulting in tubular and fragmented morphologies, respectively (Chang and Doering, 2018). However, the *mar1*Δ mutant strain again displayed a significantly larger proportion of cells with diffuse morphology (∼86%) compared to the WT strain (∼77%) and the *mar1*Δ + *MAR1* complemented strain (∼80%), and as a result displayed fewer cells with tubular and fragmented morphologies (Figure 4A, right).

To explore overall mitochondrial metabolic activity, we assessed mitochondrial mass and mitochondrial membrane potential using NAO and MitoTracker^TM^ Red CMXRos (MT), respectively. In YPD medium incubated at 30°C, the *mar1*Δ mutant strain had a modest but detectable decrease in both mitochondrial mass and mitochondrial membrane potential (NAO geometric mean = 538, MT geometric mean = 1,405) compared to both the WT strain (NAO geometric mean = 675, MT geometric mean = 1,641) and the *mar1*Δ + *MAR1* complemented strain (NAO geometric mean = 609, MT geometric mean = 1,622) (Figure 4B, top). All strains increased their mitochondrial mass and mitochondrial membrane potential in response to TC medium incubated at 37°C (Figure 4B, bottom). However, the *mar1*Δ mutant strain displayed a marked decrease in both mitochondrial mass and mitochondrial membrane potential (NAO geometric mean = 2,877, MT geometric mean = 6,067) compared to both the WT strain (NAO geometric mean = 7,215, MT geometric mean = 10,460) and the *mar1*Δ + *MAR1* complemented strain (NAO geometric mean = 6,959, MT geometric mean = 9,899) (Figure 4B, bottom).

We also measured total cellular ATP in these strains. We observed a statistically significant reduction in total cellular ATP in the *mar1*Δ mutant strain compared to the WT strain in YPD medium incubated at 30°C, which was consistent with the modest decreases in mitochondrial mass, mitochondrial membrane potential, and overall growth rate for the *mar1*Δ mutant strain in this condition (Figure 4C, top). In TC medium incubated at 37°C, all strains displayed a drastic reduction in total cellular ATP, which was expected based on the stressful nature of this condition that results in a slower growth rate for all strains (Figure 4C, top). However, the *mar1*Δ mutant strain did not reduce its total cellular ATP levels in response to host-like conditions to the same extent as the WT strain and the *mar1*Δ + *MAR1* complemented strain (Figure 4C, bottom). Collectively, these observations indicate that the *mar1*Δ mutant strain has alterations in mitochondrial metabolic activity and, as a result, Mar1 is required for normal mitochondrial homeostasis in response to host-like stress.

### Mitochondrial inhibition induces *mar1*Δ mutant strain-like cell wall phenotypes

**FIGURE 5:**
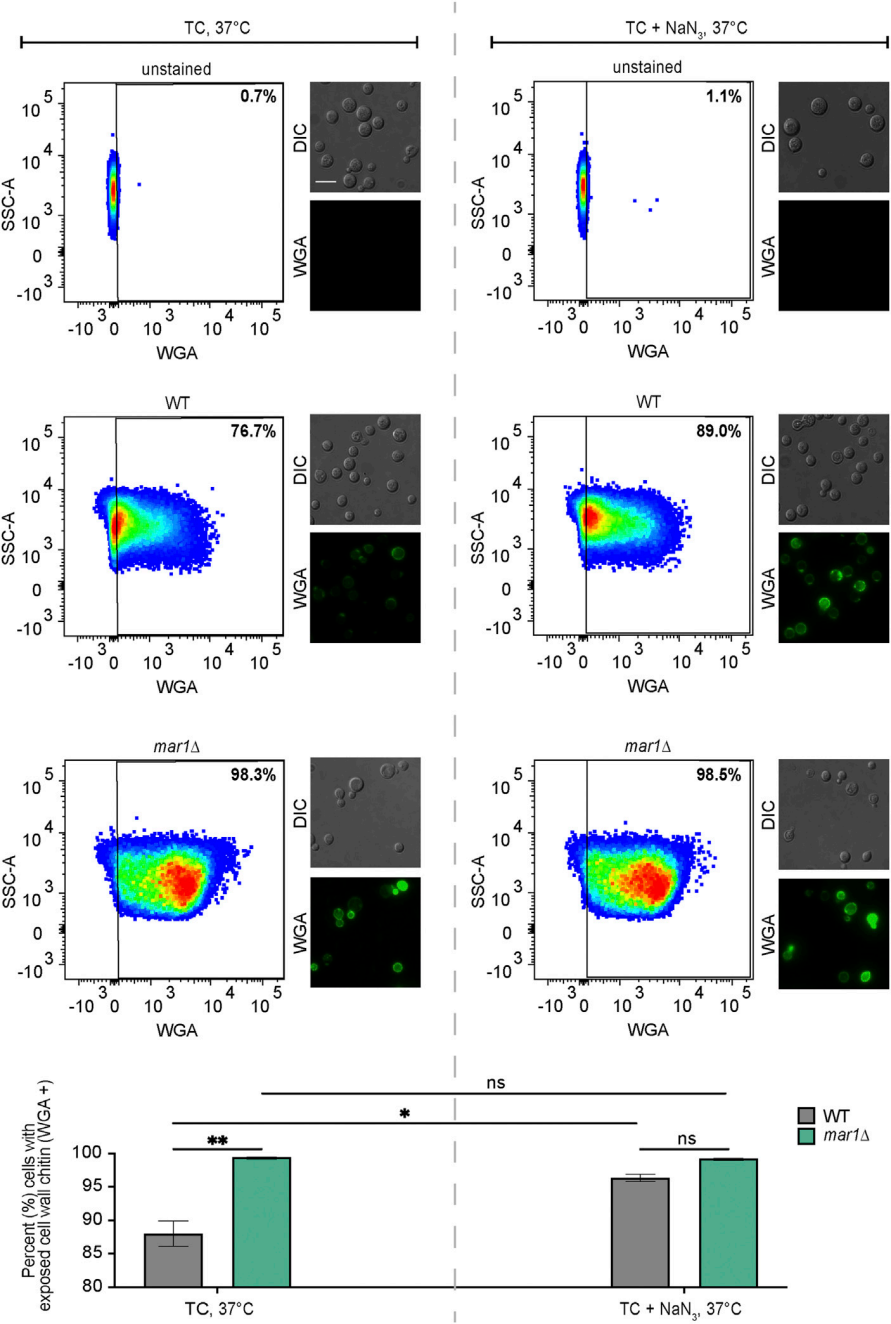
Cell wall changes induced by electron transport chain inhibition. Exposed cell wall chitin of the WT strain and the *mar1*Δ mutant strain were measured following incubation in TC medium at 37°C in the absence (left) and presence (right) of sodium azide (NaN_3_) (0.5 mM) for 24 h. Cells were stained with wheat germ agglutinin (WGA) as a marker for exposed cell wall chitin. Flow cytometry was used to quantify staining intensity. Relevant events were gated for live cells (FSC vs. SSC), single cells (FSC-H vs. FSC-A), and positive labelling (SSC vs. WGA, determined using unstained cells as negative controls). Data are represented as dot plots with mean fluorescence intensity on the x-axis and SSC-A on the y-axis, with percentages of cells displaying WGA staining included. Identical samples were also imaged by epifluorescence microscopy (63x scale bar, 10 μm). The average percentage of cells displaying WGA staining across two biological replicates (*n* = 2) is also included. Error bars represent the SEM. Statistical significance was determined using two-way ANOVA and the Tukey-Kramer test (*, *p* < 0.05; **, *p* < 0.01; ns, not significant). DIC, differential interference contrast.

Given prior reports of an association between aberrant fungal mitochondrial function and altered cell wall homeostasis, we hypothesized that the impaired mitochondrial function of the *mar1*Δ mutant strain may similarly contribute to its cell wall defects. We therefore exposed the WT strain and the *mar1*Δ mutant strain to NaN_3_, an inhibitor of electron transport chain complex IV, and quantified WGA staining as a measure of exposed cell wall chitin. Consistent with our previously published observations, the *mar1*Δ mutant strain had significantly more cells with exposed cell wall chitin compared to the WT strain in TC medium incubated at 37°C (Figure 5, left) (Esher et al., 2018). Upon 24-h exposure to sub-lethal concentrations of NaN_3_, the number of WT cells with exposed cell wall chitin increased to the level observed in the *mar1*Δ mutant strain (Figure 5, right). In contrast, the number of *mar1*Δ mutant strain cells with exposed cell wall chitin remained largely unchanged upon NaN_3_ exposure (Figure 5, right). Although these WT cells did not display the same intensity of WGA staining as the *mar1*Δ mutant cells, these results demonstrate that short-term inhibition of mitochondrial electron transport chain function is sufficient to induce *mar1*Δ mutant strain-like cell wall defects in the WT strain. This finding is supported by previous work in both *C. neoformans* (Caza et al., 2018; Horianopoulos et al., 2020) and *Candida albicans* (She et al., 2015; She et al., 2016) demonstrating that distinct mutants with impaired mitochondrial function also have cell wall defects.

### Mar1 is required for proper oxidative stress metabolism

**FIGURE 6:**
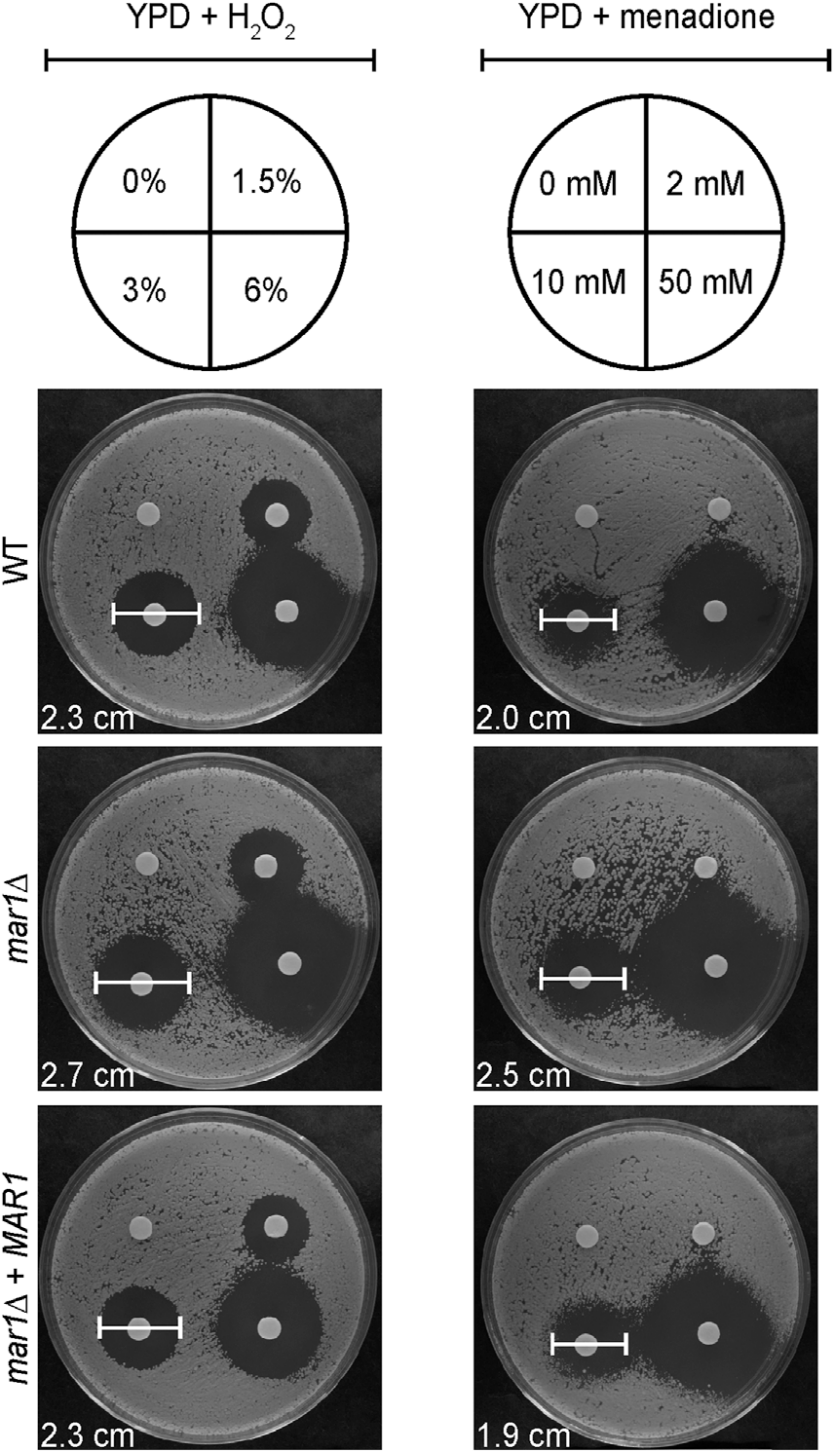
Mar1 contributions to growth in the presence of oxidative stress. A. Susceptibilities of the WT strain, the *mar1*Δ mutant strain, and the *mar1*Δ + *MAR1* complemented strain to hydrogen peroxide (H_2_O_2_) and menadione were assessed. Strains were normalized by OD_600_ and were subsequently grown on YPD medium agar plates spotted with differing concentrations of H_2_O_2_ (left) and menadione (right). Solvents alone were used as controls. Plates were incubated at 30°C and imaged daily. Zones of clearance were measured and used as indicators of susceptibility. Included zone of clearance measurements are from 3% H_2_O_2_ and 10 mM menadione. Experiment was repeated two times to ensure reproducibility and representative images are shown.

**FIGURE 7:**
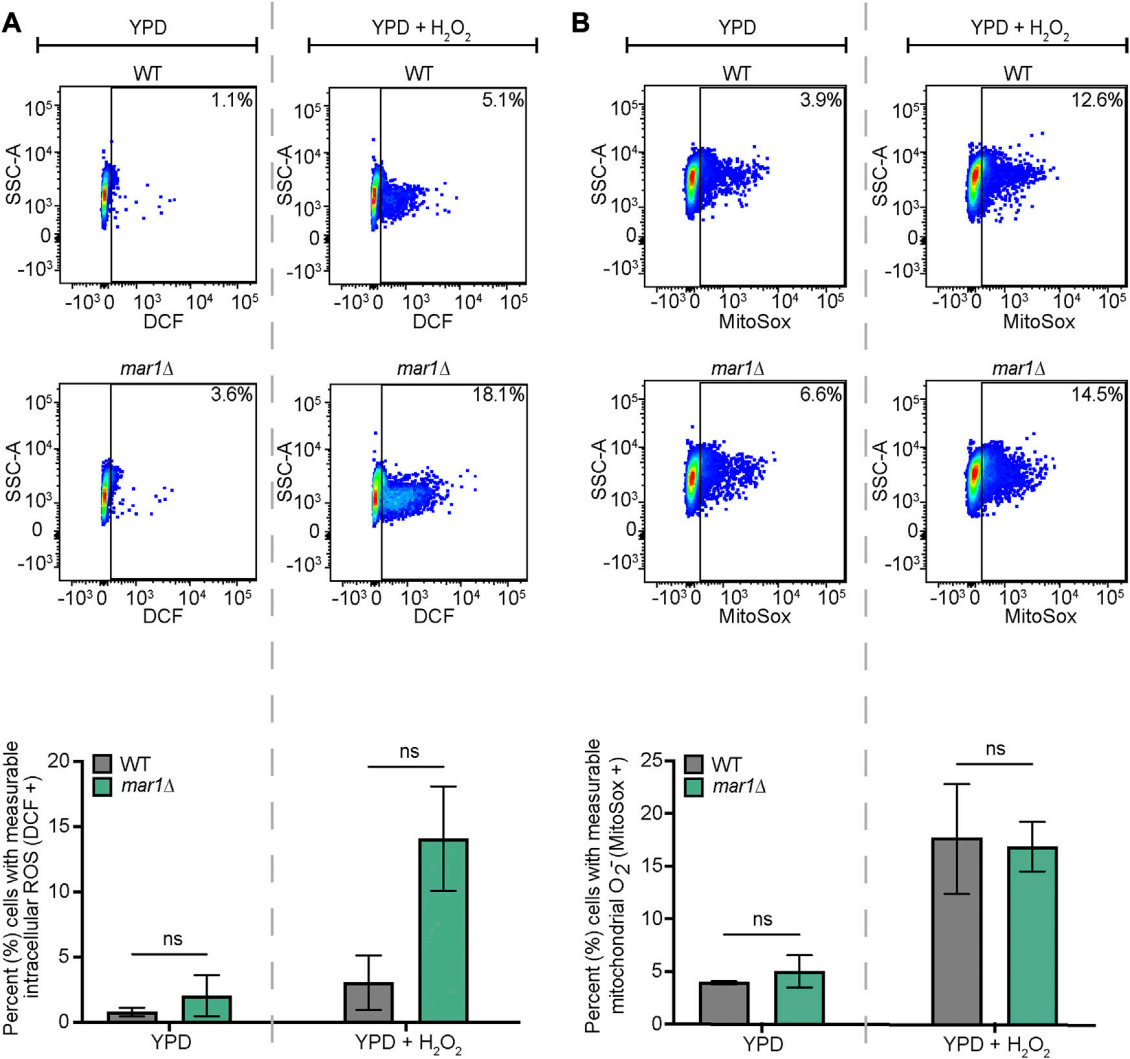
Mar1 contributions to intracellular ROS levels. **(A)** Total intracellular ROS was measured for the WT strain and the *mar1*Δ mutant strain following incubation in the absence and presence of hydrogen peroxide (H_2_O_2_) (0.01%). Cells were stained with 2′,7′-dichlorodihydrofluorescein diacetate (DCF) as a marker for intracellular ROS. Flow cytometry was used to quantify staining intensity. Relevant events were gated for live cells (FSC vs. SSC), single cells (FSC-H vs. FSC-A), and positive labelling (SSC vs. DCF, determined using unstained cells as negative controls). Data are represented as dot plots with mean fluorescence intensity on the x-axis and SSC-A on the y-axis, with percentages of cells displaying DCF staining included. The average percentage of cells displaying DCF staining across two biological replicates (*n* = 2) is also included. Error bars represent the SEM. Statistical significance was determined using two-way ANOVA and the Tukey-Kramer test (ns, not significant). **(B)** Mitochondrial superoxide was measured for the WT strain and the *mar1*Δ mutant strain following incubation in the absence and presence of hydrogen peroxide (H_2_O_2_) (0.01%). Cells were stained with MitoSox Red as a marker for mitochondrial superoxide. Flow cytometry was used to quantify staining intensity. Relevant events were gated for live cells (FSC vs. SSC), single cells (FSC-H vs. FSC-A), and positive labelling (SSC vs. MitoSox Red, determined using unstained cells as negative controls). Data are represented as dot plots with mean fluorescence intensity on the x-axis and SSC-A on the y-axis, with percentages of cells displaying MitoSox Red staining included. The average percentage of cells displaying MitoSox Red staining across two biological replicates (*n* = 2) is also included. Error bars represent the SEM. Statistical significance was determined using two-way ANOVA and the Tukey-Kramer test (ns, not significant). O_2_^−^, superoxide.

The mitochondria are major contributors of oxidative stress in the fungal cell, as well as a site in which oxidative stress is enzymatically neutralized. Because Mar1 is important for mitochondrial function particularly in the presence of host-like stress, we hypothesized that Mar1 may contribute to oxidative stress metabolism. We found that the *mar1*Δ mutant strain had increased susceptibility to both hydrogen peroxide (H_2_O_2_) and menadione compared to the WT strain and the *mar1*Δ + *MAR1* complemented strain, as measured by zones of inhibition surrounding H_2_O_2_- and menadione-containing disks (Figure 6). The WT strain and the *mar1*Δ mutant strain had similar cytosolic Sod1 protein levels and mitochondrial Sod2 protein levels, suggesting that the increased susceptibility to oxidative stress was likely not due to reduced superoxide dismutase activity (Supplementary Figure S5). We observed that the *mar1*Δ mutant strain did not have a significant increase in total intracellular ROS compared to the WT strain, as measured by 2′,7′-dichlorodihydrofluorescein diacetate (DCF) staining, in either the presence or absence of exogenous oxidative stress induced by H_2_O_2_ exposure (Figure 7A). Furthermore, we found that the *mar1*Δ mutant strain did not have a significant increase in mitochondrial superoxide, as measured by MitoSox^TM^ Red staining, in either the presence or absence of exogenous oxidative stress induced by H_2_O_2_ exposure (Figure 7B). Collectively, these observations demonstrate that Mar1 is required for proper oxidative stress metabolism.

### The *mar1*Δ mutant strain has an enhanced capacity for fluconazole tolerance

**FIGURE 8:**
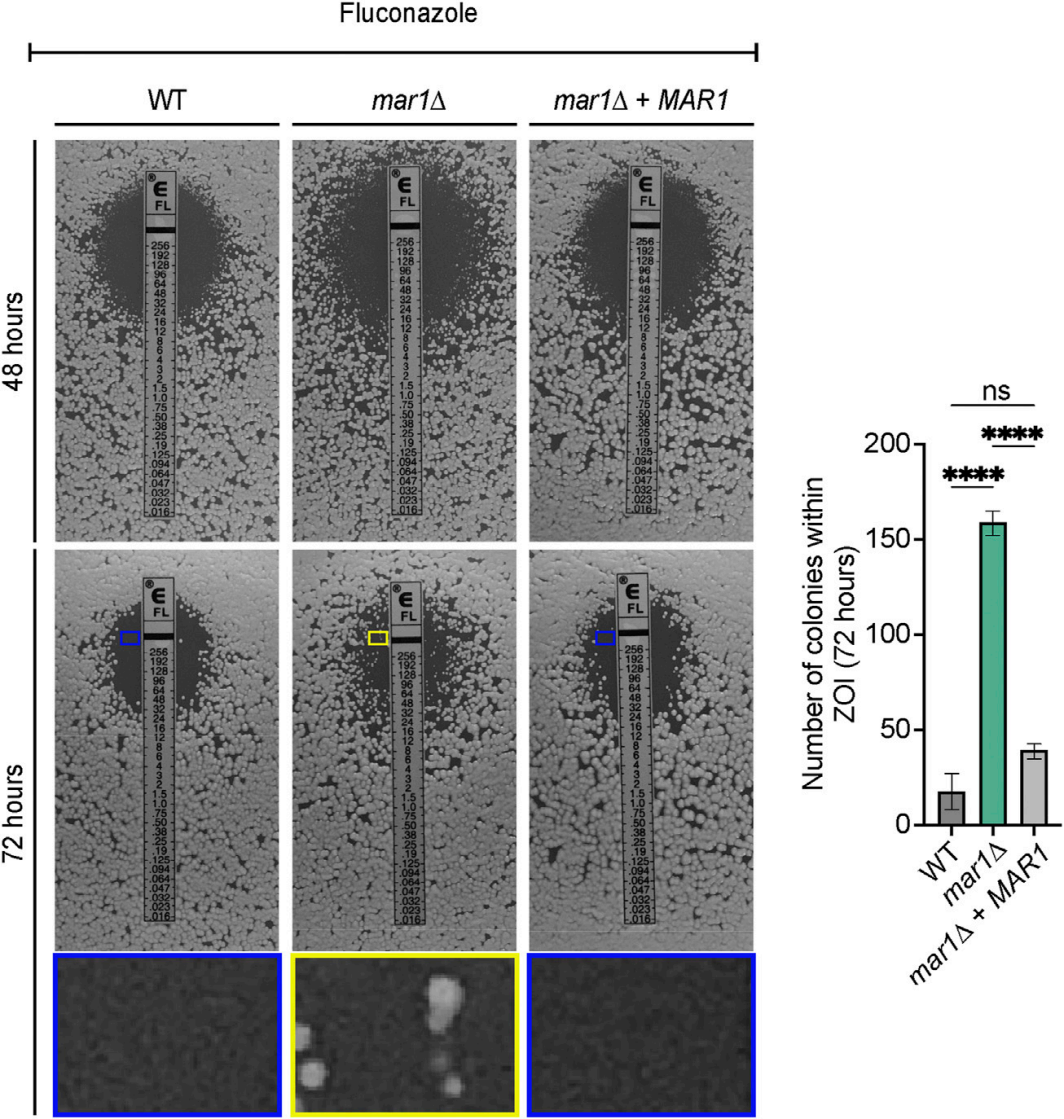
Mar1 contributions to fluconazole susceptibility. Fluconazole susceptibility and tolerance was assessed for the WT strain, the *mar1*Δ mutant strain, and the *mar1*Δ + *MAR1* complemented strain. Strains were normalized by OD_600_ and were subsequently grown on YPD medium agar plates in the presence of fluconazole Etest strips (bioMérieux). Cells were incubated at 30°C and plates were imaged at the indicated times. Fluconazole tolerance was identified by the appearance of small colonies within the zone of inhibition at 72 h. The average number of colonies growing within the zone of inhibition at 72 h for each strain across four biological replicates (*n* = 4) is also included. Error bars represent the SEM. Statistical significance was determined using one-way ANOVA and the Tukey-Kramer test (****, *p* < 0.0001; ns, not significant). ZOI, zone of inhibition. Colored boxes in the middle panels indicate inset images in the bottom panels (blue boxes = WT strain and *mar1*Δ + *MAR1* complemented strain, inside the zone of inhibition; yellow box = *mar1*Δ mutant strain, inside the zone of inhibition).

With its aberrant oxidative stress metabolism, we hypothesized that the *mar1*Δ mutant strain would exhibit altered susceptibility to antifungal drugs that induce oxidative stress, such as fluconazole. After 48 h of growth in the presence of a fluconazole gradient, all fungal strains tested displayed a clear zone of growth inhibition, demonstrating that all strains were susceptible to fluconazole in a dose-dependent manner (Figure 8, left). However, the *mar1*Δ mutant strain had a slightly lower fluconazole minimum inhibitory concentration (MIC) (12 μg/mL) than the WT strain and the *mar1*Δ + *MAR1* complemented strain (16 μg/mL), indicating that the *mar1*Δ mutant strain was slightly more susceptible to fluconazole (Figure 8, left). After 72 h of growth, many small colonies appeared within the *mar1*Δ mutant strain zone of inhibition, while the WT strain and the *mar1*Δ + *MAR1* complemented strain retained largely clear zones of inhibition (Figure 8, left). This observation was consistent across several experiments (Figure 8, right). To determine if the colonies growing within the zones of inhibition were stably fluconazole-resistant, we passaged these small *mar1*Δ mutant strain colonies to fresh YPD medium and subsequently assessed them for fluconazole susceptibility: these isolates displayed the same MIC as the original *mar1*Δ mutant strain, as well as randomly selected *mar1*Δ mutant strain colonies isolated from outside the zone of inhibition (Supplementary Figure S6). As a result, these small *mar1*Δ mutant strain colonies were not stably resistant to fluconazole but were reminiscent of fluconazole “tolerant” colonies previously identified and characterized in *Candida* species (Rosenberg et al., 2018; Berman and Krysan, 2020). These fluconazole-tolerant *mar1*Δ mutant strain colonies contrasted starkly with a large WT strain colony isolated from within the WT strain zone of inhibition, which was determined to be stably fluconazole resistant after passaging to fresh YPD medium: this isolate demonstrated an increased MIC compared to the original WT strain, as well as compared to randomly selected colonies from outside the zone of inhibition (Supplementary Figure S6). These observations demonstrate that the *mar1*Δ mutant strain has an enhanced capacity to develop fluconazole tolerance.

**FIGURE 9:**
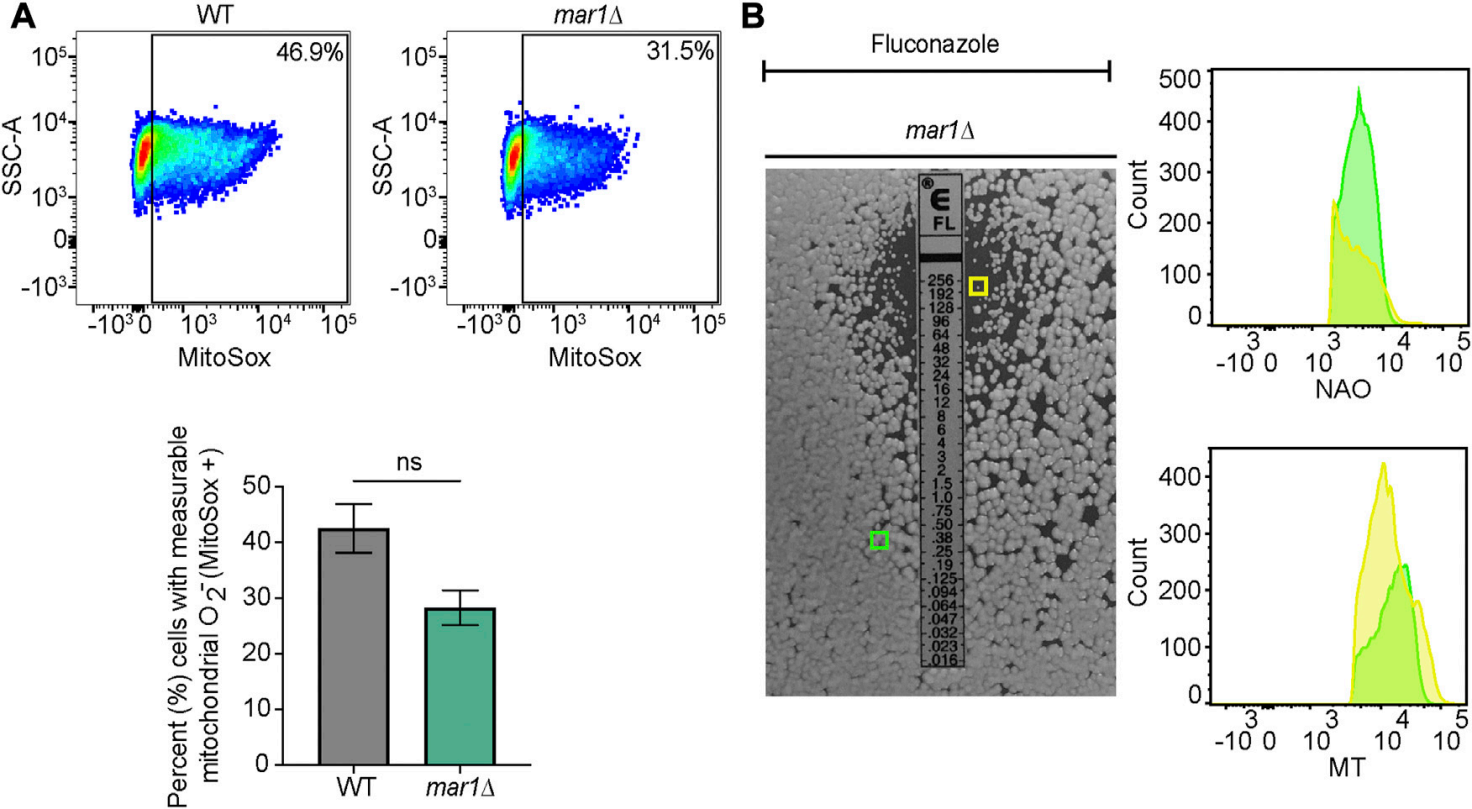
Mitochondrial phenotypes associated with fluconazole tolerance. **(A)** Mitochondrial superoxide was measured for the WT strain and the *mar1*Δ mutant strain following incubation in the presence of fluconazole (10 μg/mL). Cells were stained with MitoSox Red as a marker for mitochondrial superoxide. Flow cytometry was used to quantify staining intensity. Relevant events were gated for live cells (FSC vs. SSC), single cells (FSC-H vs. FSC-A), and positive labelling (SSC vs. MitoSox Red, determined using unstained cells as negative controls). Data are represented as dot plots with mean fluorescence intensity on the x-axis and SSC-A on the y-axis, with percentages of cells displaying MitoSox Red staining included. The average percentage of cells displaying MitoSox Red staining across two biological replicates (*n* = 2) is also included. Error bars represent the SEM. Statistical significance was determined using Student's *t* test (ns, not significant). O_2_^−^, superoxide. **(B)** Mitochondrial mass and mitochondrial membrane potential of *mar1*Δ mutant strain fluconazole-tolerant and non-tolerant cells from the assay described in Figure 8 were measured. Tolerant (within the zone of inhibition) and non-tolerant (outside of the zone of inhibition) colonies were isolated and mitochondria were co-stained with nonyl acridine orange (NAO), as a marker of mitochondrial mass, MitoTracker^TM^ Red CMXRos (MT), as a marker of mitochondrial membrane potential, and Sytox^TM^ Blue (SB). Flow cytometry was used to quantify staining intensity. Relevant events were gated for live cells (FSC vs. SSC and SSC vs. SB), single cells (FSC-H vs. FSC-A), and positive labelling (SSC vs. NAO/MT, determined using unstained cells as negative controls). Data are represented as histograms with mean fluorescence intensity on the x-axis and cell number of the y-axis. Experiment was repeated twice to ensure reproducibility. Colored boxes indicate colonies assessed in histograms (yellow box = *mar1*Δ mutant strain colony isolated from inside the zone of inhibition; green box = *mar1*Δ mutant strain colony isolated from outside the zone of inhibition).

Previously published work has demonstrated that *C. neoformans* exposure to fluconazole results in increased levels of total intracellular ROS (Peng et al., 2018; Dbouk et al., 2019). We found that fluconazole exposure also resulted in increased levels of mitochondrial superoxide in WT *C. neoformans*; less than 5% of WT cells displayed measurable mitochondrial superoxide in YPD alone (Figure 7B) while over 40% of WT cells displayed measurable mitochondrial superoxide upon exposure to fluconazole (Figure 9A). No significant differences in mitochondrial superoxide were observed between the WT strain and the *mar1*Δ mutant strain upon fluconazole exposure (Figure 9A). However, using the *mar1*Δ mutant strain as a model for fluconazole tolerance in *C. neoformans*, we found that fluconazole-tolerant *mar1*Δ mutant strain cells had distinct mitochondrial metabolic activity profiles compared to non-tolerant *mar1*Δ mutant strain cells. Fluconazole-tolerant *mar1*Δ mutant strain cells consistently displayed decreased mitochondrial mass and mitochondrial membrane potential (NAO geometric mean = 4,181, MT geometric mean = 13,737) compared to non-tolerant *mar1*Δ mutant strain cells (NAO geometric mean = 4,344, MT geometric mean = 14,868) (Figure 9B). These data suggest that modest alterations in mitochondrial metabolic activity may contribute to fluconazole tolerance in *C. neoformans*.

## Discussion

### Mar1 is a mitochondrial localized protein required for normal mitochondrial metabolic activity

We previously demonstrated that the Mar1 protein is required for normal cell wall remodeling in response to host-like conditions (Esher et al., 2018). The implications of Mar1-mediated cell wall remodeling on pathogenesis are significant. The *mar1*Δ mutant strain is hypovirulent in a murine model, likely as a result of enhanced macrophage activation due to its immunogenic cell surface (Esher et al., 2018). Additionally, inoculation with the *mar1*Δ mutant strain results in the establishment of stable murine lung granulomas, creating a persistent infection that more closely resembles the normal course of cryptococcal disease in humans, as opposed to the more aggressive proliferation in the lung followed by systemic dissemination observed in primary murine infections with the WT strain (Telzrow et al., 2022). In this work, we explored the molecular mechanisms by which Mar1 contributes to fungal cell wall remodeling.

Mar1 is a *Cryptococcus*-specific protein lacking identifiable orthologs in other fungi (Esher et al., 2018). We used an RNA sequencing approach to infer cellular processes that are affected by Mar1. We found that in host-like conditions, the *mar1*Δ mutant strain displayed abnormal transcriptional profiles of many genes predicted to localize to the mitochondria and known to contribute to mitochondrial function, including genes involved in carbohydrate metabolism and transport. We previously reported that the *mar1*Δ mutant strain has decreased cell wall glucan and mannan (Esher et al., 2018). Transcriptional alterations in carbohydrate metabolism and import may, in part, explain these *mar1*Δ mutant strain cell wall defects, as carbon source type and availability strongly influence fungal cell wall structure (Ene et al., 2015).

Subcellular fractionation analyses determined that Mar1 localized to the mitochondria. Because Mar1 is a nuclear-encoded protein, it must be imported into the mitochondria from the cytosol following translation. Mar1 does not contain an N-terminal presequence, which targets proteins to the mitochondrial matrix. This suggests that Mar1 likely localizes to the outer mitochondrial membrane, the intermembrane space, or the inner mitochondrial membrane, because nuclear-encoded proteins that lack presequences typically localize to these areas (Chacinska et al., 2002). The only annotated domain within the Mar1 protein is a domain of unknown function (DUF4112) which harbors two transmembrane domains (Esher et al., 2018). The *C. neoformans* proteome contains multiple mitochondrial membrane proteins that contain two transmembrane domains, including solute carrier (SLC) family 25 proteins (CNAG_03824, CNAG_05283, and CNAG_01808), translocase of the inner membrane (TIM) proteins (CNAG_04287), and presequence translocated-associated motor (PAM) proteins (CNAG_07720). This shared structure suggests that Mar1 may be involved in the transport of proteins, lipids, and/or metabolites between the cytosol and the mitochondria. Furthermore, in addition to Mar1, there are only two proteins in the *C. neoformans* proteome that contain DUF4112: CNAG_01821 and CNAG_04386. Like Mar1, both proteins are uncharacterized, harbor two transmembrane domains within DUF4112, and have a relatively high probability of mitochondrial import by MitoProt II prediction (CNAG_01821 = 72%, CNAG_04386 = 57%) (Claros and Vincens, 1996). It is possible that these proteins comprise an uncharacterized family of mitochondrial localized proteins.

Our characterization of Mar1 as a mitochondrial localized protein suggested that Mar1 might contribute to mitochondrial function. We found that Mar1 was required for normal electron transport chain function. Compared to growth on YPD medium alone, the *mar1*Δ mutant strain displayed enhanced growth defects in the presence of complex II, III, and IV inhibitors, but not inhibitors of complex I and the alternative oxidase. In eukaryotes, NADH is the main electron carrier in oxidative phosphorylation, in which electrons flow from NADH → complex I → complex III → complex IV → O_2_ (Ahmad et al., 2022). The other electron carrier, FADH_2_, passes electrons from FADH_2_ → complex II → complex III → complex IV → O_2_ (Ahmad et al., 2022). One hypothesis for the specificity of electron transport chain phenotypes observed is that the *mar1*Δ mutant strain relies more heavily on FADH_2_ as an electron carrier than NADH, and as a result utilizes electron flow largely through complexes II, III, and IV and not complex I. A resulting decreased availability of NADH in *mar1*Δ mutant strain mitochondria could underlie this phenotype. In *S. cerevisiae*, there are many shuttles involved in maintaining NADH within the mitochondria, including the malate-aspartate shuttle, which translocates electrons produced during glycolysis to the mitochondrial matrix to form NADH (Cavero et al., 2003), and the mitochondrial NAD^+^ carrier proteins, which shuttle cytosolic NAD^+^ into the mitochondria to produce NADH in the TCA cycle (Todisco et al., 2006). The presence of transmembrane domains within the Mar1 protein supports a potential role of Mar1 as a mitochondrial carrier protein (Palmieri, 2004). Alternatively, reliance on electron flow through complex II could be a result of structural defects in complex I. Mammalian complex I is made up of 44 protein components, 37 of which are encoded in the nuclear genome (Smeitink et al., 1998; Voet et al., 2013) and are imported into the mitochondria with the help of many protein chaperones and translocases. If Mar1 functions as a mitochondrial chaperone or mitochondrial membrane translocase, the *mar1*Δ mutant strain may be unable to import and assemble all the necessary components of complex I into the mitochondria. Even though the electron transport chain is a shared characteristic of eukaryotes, the proteins that comprise the electron transport chain display evolutionary divergence. For example, Goa1 is a *Candida*-specific protein that is required for the assembly of complex I in *C. albicans* (Bambach et al., 2009). Therefore, it is additionally possible that Mar1 could be a *Cryptococcus*-specific component of complex I.

In contrast to the previously characterized *kcs1*Δ mutant strain, the *mar1*Δ mutant strain did not appear to have a defect in carbon source utilization. Compared to growth on YPD medium alone, the *mar1*Δ mutant strain did not demonstrate exacerbated growth defects in carbon deprivation or growth with galactose, glycerol, or acetate as the only available carbon sources. However, the *mar1*Δ mutant strain displayed enhanced growth defects when utilizing ethanol and butyric acid as sole carbon sources, phenotypes that were incompletely rescued by supplementation of the preferred carbon source glucose. These observations suggest that these growth attenuation phenotypes are, at least in part, due to the accumulation of toxic intermediates, such as acetaldehyde in the case of growth in the presence of ethanol (Jones, 1990) and fatty acids in the case of growth in the presence of butyric acid (Guimarães and Venâncio, 2022), and not simply carbon deprivation caused by a general defect in alternative carbon source utilization. In future work, it will be important to test the ability of the *mar1*Δ mutant strain to use other fatty acids (saturated and unsaturated) as sole carbon sources and the effects of glucose supplementation in these cases, to put the butyric acid growth effects in this work into a larger context. Collectively, these data suggest that the *mar1*Δ mutant strain does not have a major metabolic shift that would necessitate detailed metabolic analyses.

With the knowledge that the *mar1*Δ mutant strain had inherent defects in electron transport chain function, we explored *mar1*Δ mutant strain mitochondrial homeostasis in both permissive and stressful growth conditions. We observed that the *mar1*Δ mutant strain displayed diffuse mitochondrial morphology more frequently than the WT strain, in both YPD medium incubated at 30°C and TC medium incubated at 37°C. In yeast, including *Cryptococcus* species, mitochondria exist primarily in the diffuse morphology during permissive, non-stressful growth (Chan, 2006; Chang and Doering, 2018). Upon mitochondrial stress, mitochondria undergo fusion and fission, resulting in enrichment of tubular and fragmented morphologies, respectively (Chang and Doering, 2018). Mitochondrial fusion allows for the sharing of mitochondrial DNA, while mitochondrial fission facilitates the removal of damaged mitochondria. The increase in diffuse morphology in the *mar1*Δ mutant strain, and the concomitant decreases in tubular and fragmented morphologies, suggest that this strain is not properly adapting to mitochondrial stress. Multiple studies have demonstrated the importance of mitochondrial morphology for fitness and virulence in human fungal pathogens, including *Cryptococcus* species. In *C. gattii*, enhanced tubular mitochondrial morphology was found to promote virulence (Ma et al., 2009). In *C. neoformans*, the ability to undergo mitochondrial fusion and fission is required for growth in the presence of oxidative stress, elaboration of virulence factors, and virulence (Chang and Doering, 2018). Because the tubular and fragmented morphologies were not completely absent in the *mar1*Δ mutant strain, Mar1 is not absolutely required for mitochondrial fission and fusion.

We further found that the *mar1*Δ mutant strain had a reduction in mitochondrial mass and mitochondrial membrane potential compared to the WT strain, a phenotype observed in YPD medium incubated at 30°C but markedly enhanced in TC medium incubated at 37°C. The WT strain drastically increased both its mitochondrial mass and mitochondrial membrane potential in response to incubation in TC medium at 37°C, likely as a result of mitochondrial biogenesis. Mitochondrial biogenesis is a common response to mitochondrial stress (Lee and Wei, 2005) and involves the import of many proteins, lipids, and metabolites into the mitochondria (Diaz and Moraes, 2008). Although the *mar1*Δ mutant strain did increase both its mitochondrial mass and mitochondrial membrane potential in response to incubation in TC medium at 37°C, it was unable to do so to the level of the WT strain.

Both the mitochondrial morphology and mitochondrial mass/membrane potential phenotypes suggest that the *mar1*Δ mutant strain does not sense mitochondrial stress as readily as the WT strain. As a *Cryptococcus*-specific protein lacking domains indicative of function, Mar1 could be serving as a stress sensor. We previously reported that Mar1 also localizes in punctate structures on the cell surface (Esher et al., 2018). It is possible that Mar1 is responsible for sensing extracellular stress and communicating stress signals to the mitochondria. Alternatively, it is possible that the *mar1*Δ mutant strain is limited in the amount of mitochondrial fission, fusion, or biogenesis that it can perform, potentially because of a limitation in proteins, lipids, or metabolites required for these processes in the mitochondria.

The reduction in mitochondrial mass and mitochondrial membrane potential of the *mar1*Δ mutant strain could explain the significant decrease in total cellular ATP observed in YPD medium incubated at 30°C. Upon exposure to TC medium incubated at 37°C, we observed that all strains had a drastic reduction in total cellular ATP. However, the *mar1*Δ mutant strain appeared to have a slight increase in total cellular ATP compared to the WT strain in this condition. It is possible that this phenotype is due to reduced ATP consumption of the *mar1*Δ mutant strain in the presence of cell stress. Alternatively, as suggested by our previous mitochondrial morphology and mitochondrial mass/membrane potential phenotypes, it is possible that the *mar1*Δ mutant strain is impaired in its sensing of mitochondrial stress in TC medium incubated at 37°C, and as a result does not reduce its ATP synthesis as rapidly as the WT strain.

### Mar1-regulated mitochondrial processes have diverse physiological effects

We previously reported that the *mar1*Δ mutant strain has severe cell wall remodeling defects in TC medium incubated at 37°C, including an aberrant increase in exposed cell wall chitin (Esher et al., 2018). We found that chemical inhibition of complex IV in TC medium at 37°C resulted in *mar1*Δ mutant strain-like cell wall defects in the WT strain, specifically more cells with exposed cell wall chitin. Therefore, inhibition of mitochondrial function in host-like conditions is sufficient to induce similar disordering of the *C. neoformans* cell wall as observed in the *mar1*Δ mutant strain. Previous investigations, largely in *Candida* species, revealed that mitochondrial function is important for normal cell wall integrity and remodeling due to mitochondrial contributions to carbohydrate metabolism, lipid metabolism, and respiration (Koch et al., 2019). This connection has been newly explored in *Cryptococcus* species, in which investigators noted that mutants with mitochondrial defects are susceptible to cell wall targeting agents (Caza et al., 2018; Horianopoulos et al., 2020), and that electron transport chain inhibition results in changes in cell wall structure (Horianopoulos et al., 2020).

In addition to direct mitochondrial defects, we found that the *mar1*Δ mutant strain displayed alterations in mitochondria-related functions, including responses to oxidative stress. Specifically, the *mar1*Δ mutant strain had enhanced susceptibility to exogenous oxidative stress. Intact mitochondrial function is important for the clearance of ROS, largely through the function of SOD enzymes. In *C. neoformans*, Sod1 primarily localizes to the cytoplasm while Sod2 primarily localizes to the mitochondrial matrix (Smith et al., 2021). We found that the *mar1*Δ mutant strain had comparable Sod1 and Sod2 proteins levels to the WT strain, demonstrating that its susceptibility to oxidative stress is likely not caused by reduced SOD activity. Because the *mar1*Δ mutant strain has defects in electron transport chain function, it is possible that it is experiencing enhanced electron leakage from the electron transport chain. For example, if Mar1 is involved in complex I assembly, a hypothesis suggested by our electron transport chain inhibition experiments, dysfunction of complex I in the *mar1*Δ mutant strain could cause electron leakage. This hypothesis is supported by work in *C. albicans*, in which inactivation of complex I protein Goa1 leads to increased intracellular ROS (She et al., 2013).

**FIGURE 10:**
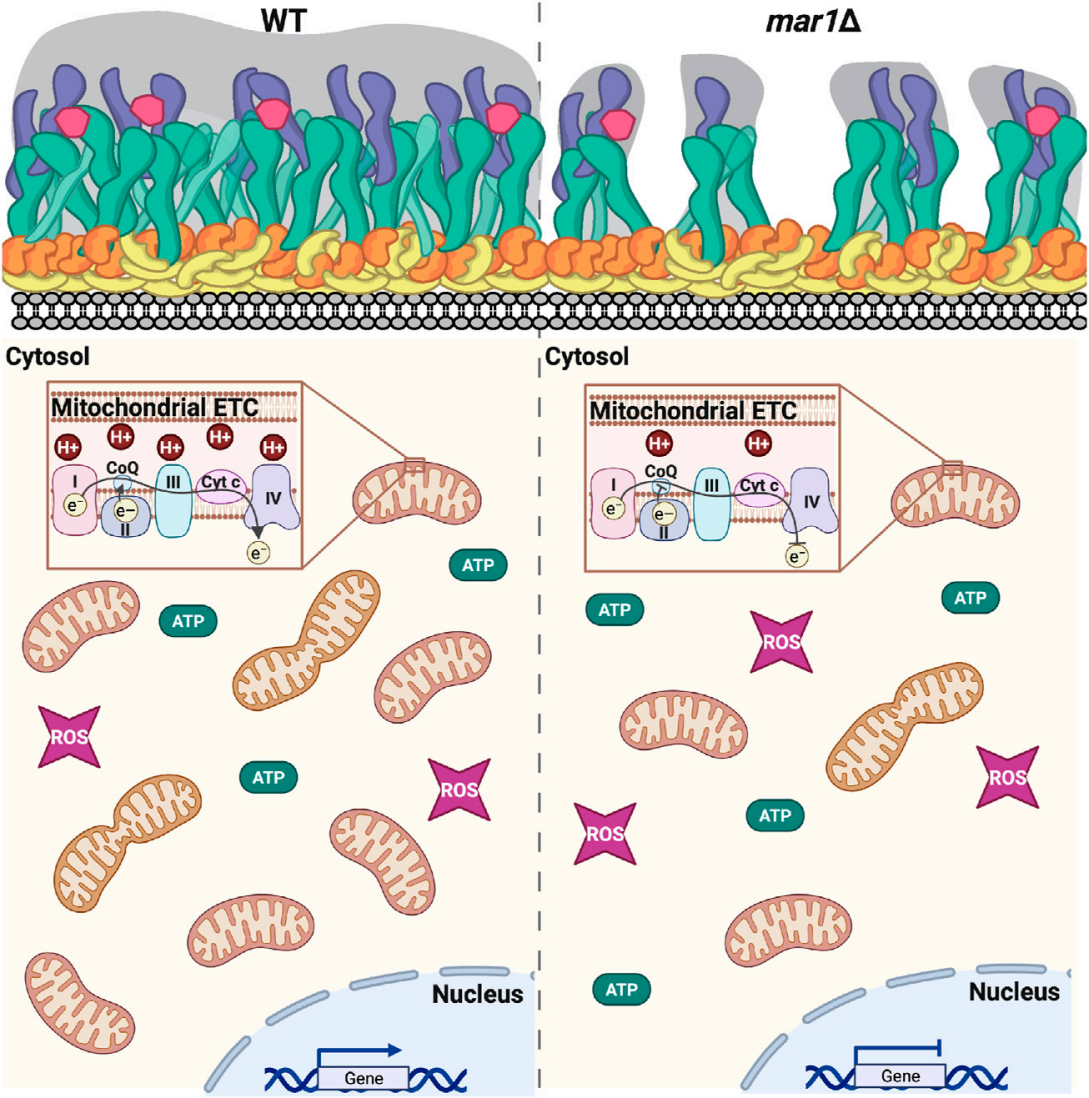
Working model of *mar1*Δ mutant strain phenotypes. As previously described, in response to host-like stressors, the WT strain remodels its cell surface by thickening its cell wall and extending its polysaccharide capsule (Esher et al., 2018). In this work, we find that the WT strain also adapts to these conditions by altering expression of nuclear-encoded genes involved in mitochondrial function increasing its mitochondrial mass, increasing its mitochondrial membrane potential, and reducing its total cellular ATP. The *mar1*Δ mutant strain is impaired in these adaptive cellular responses to host-like conditions. As previously described, compared to the WT strain, the *mar1*Δ mutant strain has attenuation in cell wall thickening; the *mar1*Δ mutant strain displays increased chitin exposure likely due to decreased levels of glucans and mannans in the outer cell wall layers and subsequent decreased polysaccharide capsule extension in response to host-like stressors (Esher et al., 2018). In this work, we find that the *mar1*Δ mutant strain has aberrant expression of nuclear-encoded genes involved in mitochondrial function, decreased mitochondrial mass, decreased mitochondrial membrane potential, and altered ATP metabolism. Collectively, these data implicate Mar1 in regulation of mitochondrial metabolism and homeostasis, particularly in the presence of stress. Cartoon adapted from BioRender.com (2022). Cell surface components: yellow = chitin; orange = chitosan; green = β-glucans; purple = α-glucans; pink = mannoproteins; gray = polysaccharide capsule. ETC., electron transport chain. CoQ, coenzyme Q. Cyt c, cytochrome c. ROS, reactive oxygen species.

Based on previous work implicating mitochondrial function in cell surface remodeling, we propose that Mar1-dependent modulation of mitochondrial metabolism and homeostasis regulates cell surface remodeling in response to host-like stress (Figure 10). The hypotheses of Mar1 function presented above will be important to explore to better understand the mechanisms by which Mar1 contributes to mitochondrial function and cell wall remodeling.

### Antifungal tolerance in associated with repressed mitochondrial activity in *C. neoformans*

With its alterations in oxidative stress metabolism, we hypothesized that the *mar1*Δ mutant strain would exhibit altered susceptibility to antifungal drugs that induce oxidative stress. Fluconazole is a clinically relevant antifungal that targets ergosterol biosynthesis. It has been shown to cause ROS accumulation in *Cryptococcus* species which is, in part, responsible for its inhibitory activity (Peng et al., 2018; Dbouk et al., 2019). Although the *mar1*Δ mutant strain had a slight increase in susceptibility to fluconazole after 48 h of exposure, it displayed an enhanced capacity for fluconazole tolerance by 72 h of exposure. The definitions, history, and clinical importance of antifungal tolerance were recently summarized (Berman and Krysan, 2020). While true fluconazole resistance is usually driven by genetic mutations, fluconazole tolerance is caused by epigenetic and non-genetic mechanisms, such as metabolic heterogeneity within an isogenic population of fungal cells (Rosenberg et al., 2018; Berman and Krysan, 2020). It should be noted that we cannot completely rule out the possibility that the *mar1*Δ mutant strain was developing heteroresistance to fluconazole, rather than tolerance, because heteroresistance is a common mechanism of response by *Cryptococcus* species to antifungals (Sionov et al., 2009; Berman and Krysan, 2020). However, the fact that the *mar1*Δ mutant strain colonies growing within the zone of inhibition were smaller than those growing outside of the zone of inhibition, as well as the fact that *mar1*Δ mutant strain colony growth within the zone of inhibition was independent of fluconazole concentration, support their characterization as tolerant, rather than heteroresistant (Berman and Krysan, 2020).

Previously, two independent groups demonstrated that exposure of *C. neoformans* to fluconazole results in increased levels of total intracellular ROS (Peng et al., 2018; Dbouk et al., 2019). Both groups also demonstrated that exogenous antioxidants resulted in improved fungal growth in the presence of fluconazole (Peng et al., 2018; Dbouk et al., 2019). Furthermore, Peng *et al.* suggested that fluconazole mediates DNA damage and chromosomal instability by associating with metals such as Cu and Fe and favoring oxidation states that lead to ROS production (Peng et al., 2018). Our observation that there were no significant differences in mitochondrial superoxide accumulation between the WT strain and the *mar1*Δ mutant strain in the presence of fluconazole suggests that the enhanced azole tolerance observed in the *mar1*Δ mutant strain is not induced by mitochondrial oxidative stress. In contrast, increased antifungal tolerance may be associated with repressed mitochondrial metabolic activity. Studies in *Staphylococcus aureus* have found that reduced bacterial respiration and overall metabolic activity promote antibiotic tolerance (Rowe et al., 2020). It is therefore possible that mitochondrial metabolic activity in fungi similarly drives the capacity for antifungal tolerance. This hypothesis is consistent with our observation that fluconazole-tolerant *mar1*Δ mutant strain cells had decreased mitochondrial mass and mitochondrial membrane potential compared to non-tolerant cells.

In addition to its ability to persist in the presence of inhibitory concentrations of fluconazole, we previously reported that the *mar1*Δ mutant strain can also persist long-term in the mammalian lung environment within granulomas (Telzrow et al., 2022). Mitochondrial metabolic activity has recently been demonstrated to contribute to *C. neoformans* persistence both *in vitro* and *in vivo* (Alanio et al., 2015; Hommel et al., 2019). Considering these observations, it will be important to explore the mitochondrial metabolism of the *mar1*Δ mutant strain within this host niche, particularly *mar1*Δ mutant strain cells that persist within granulomas. Studies like this can help us understand the roles of fungal mitochondrial metabolic activity in stress tolerance, including tolerance to the mammalian host.

## Data Availability

The RNA sequencing data analyzed in this publication have been deposited in NCBI’s Gene Expression Omnibus (GEO) (30,31) and are accessible through GEO Series accession number GSE160397 (https://www.ncbi.nlm.nih.gov/geo/query/acc.cgi?acc=GSE160397).
