# Supplementary figures and images for: Cryptococcus neoformans Mar1 function links mitochondrial metabolism, oxidative stress, and antifungal tolerance
Source: Front Physiol. 2023 Mar 9;14:1150272. doi: 10.3389/fphys.2023.1150272 (PMC10033685; doi:10.3389/fphys.2023.1150272)

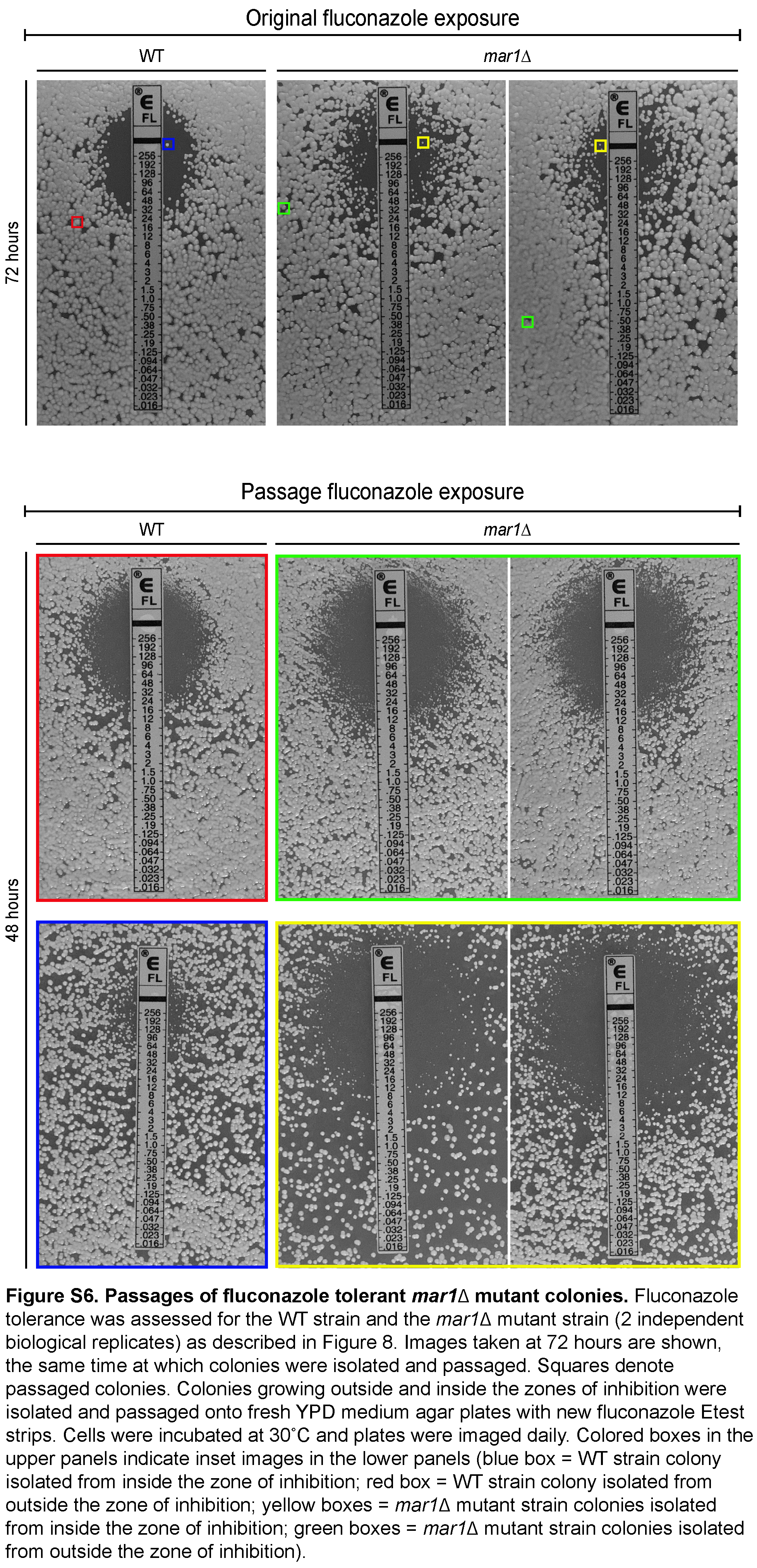

Supplement: Supplementary file 3 [file Image6.TIF]

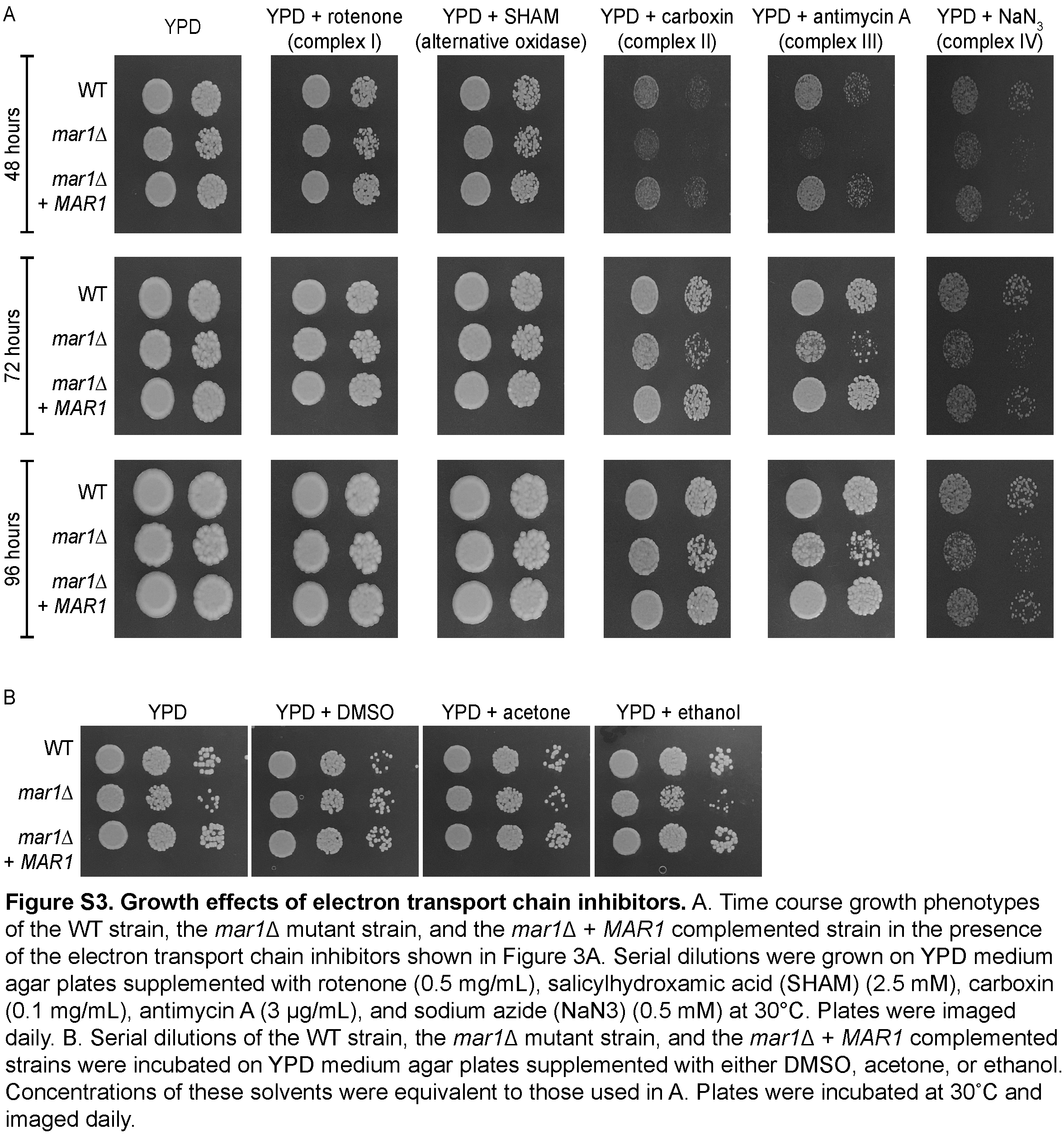

Supplement: Supplementary file 4 [file Image3.TIF]

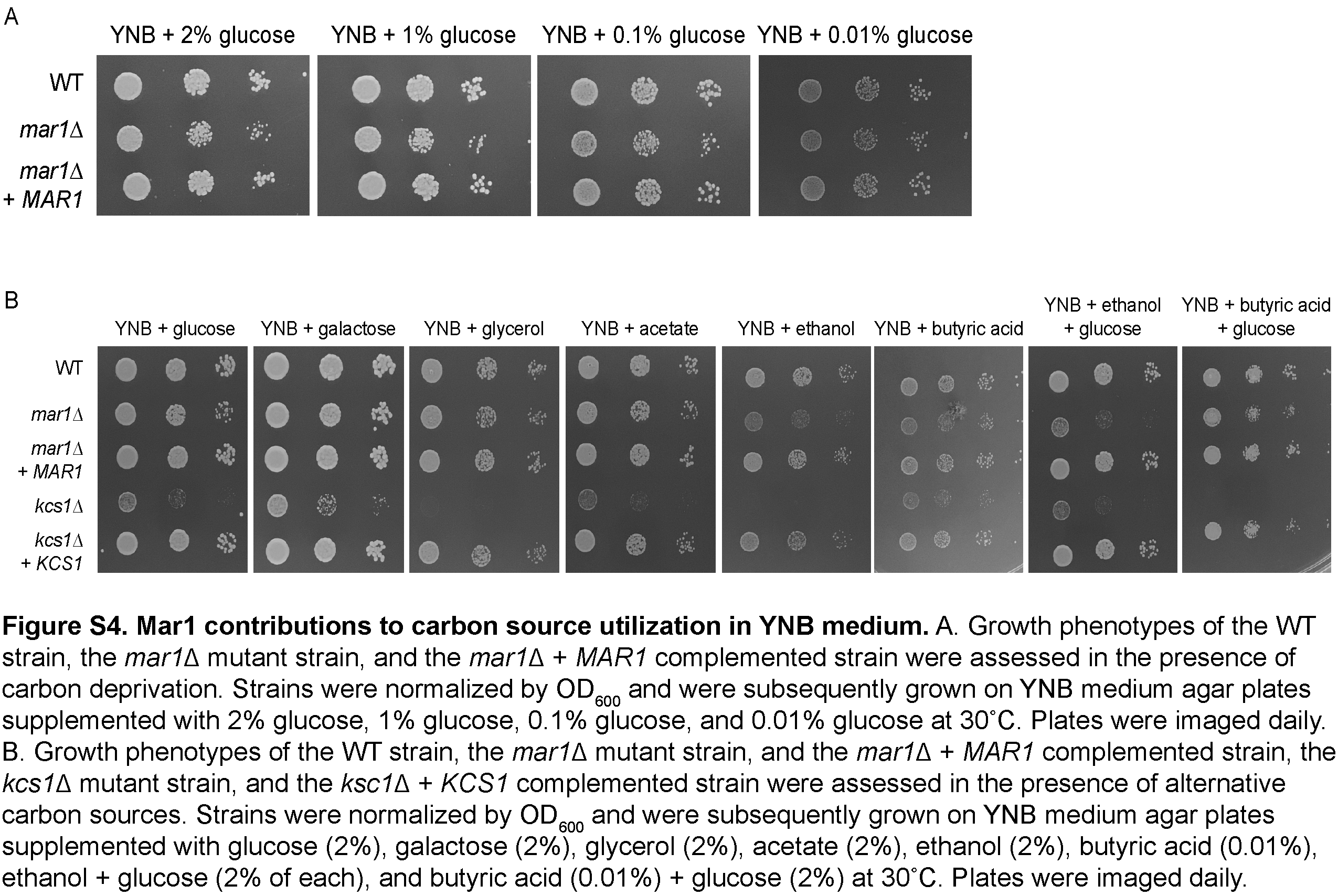

Supplement: Supplementary file 5 [file Image4.TIF]

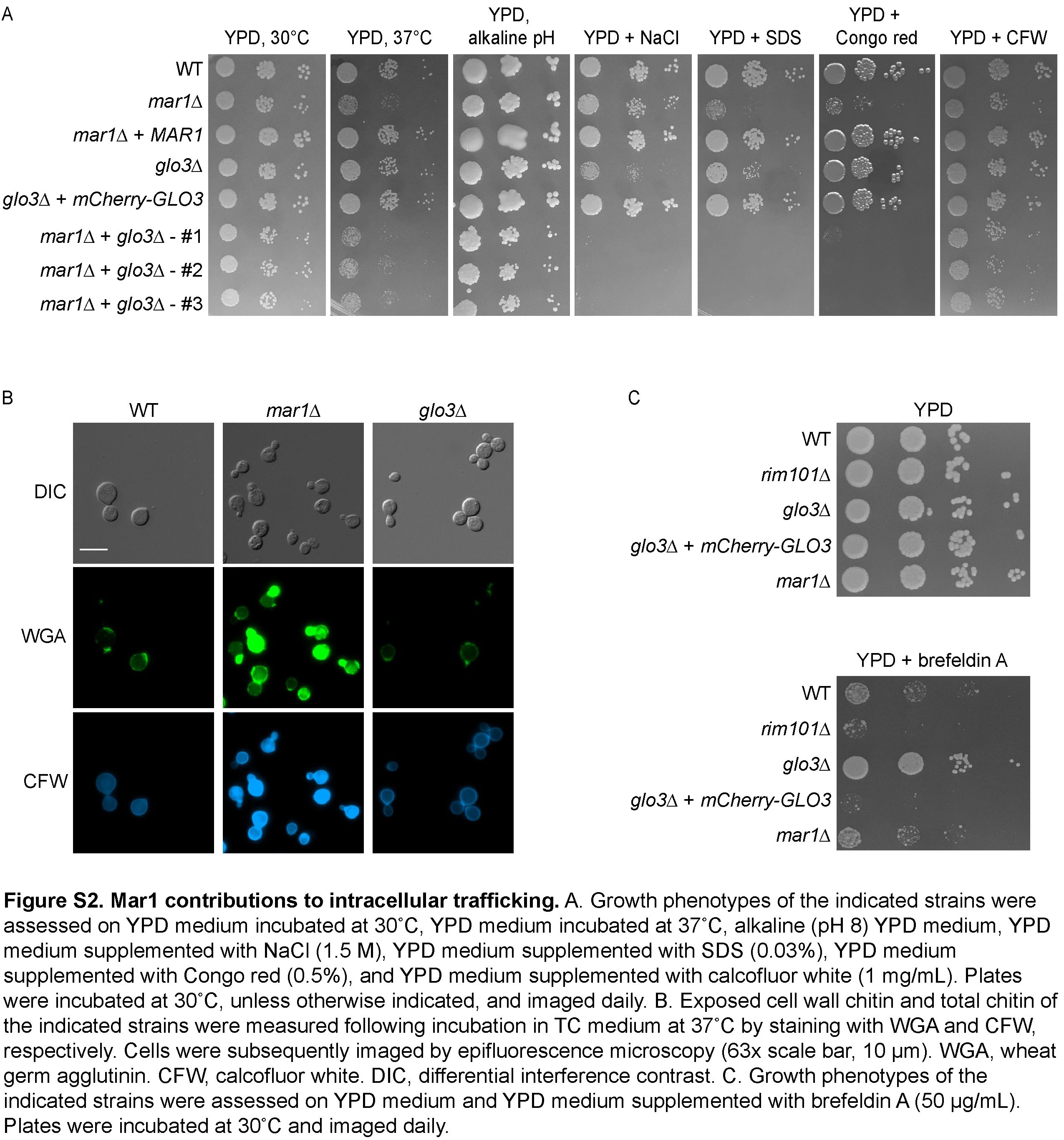

Supplement: Supplementary file 6 [file Image2.TIF]

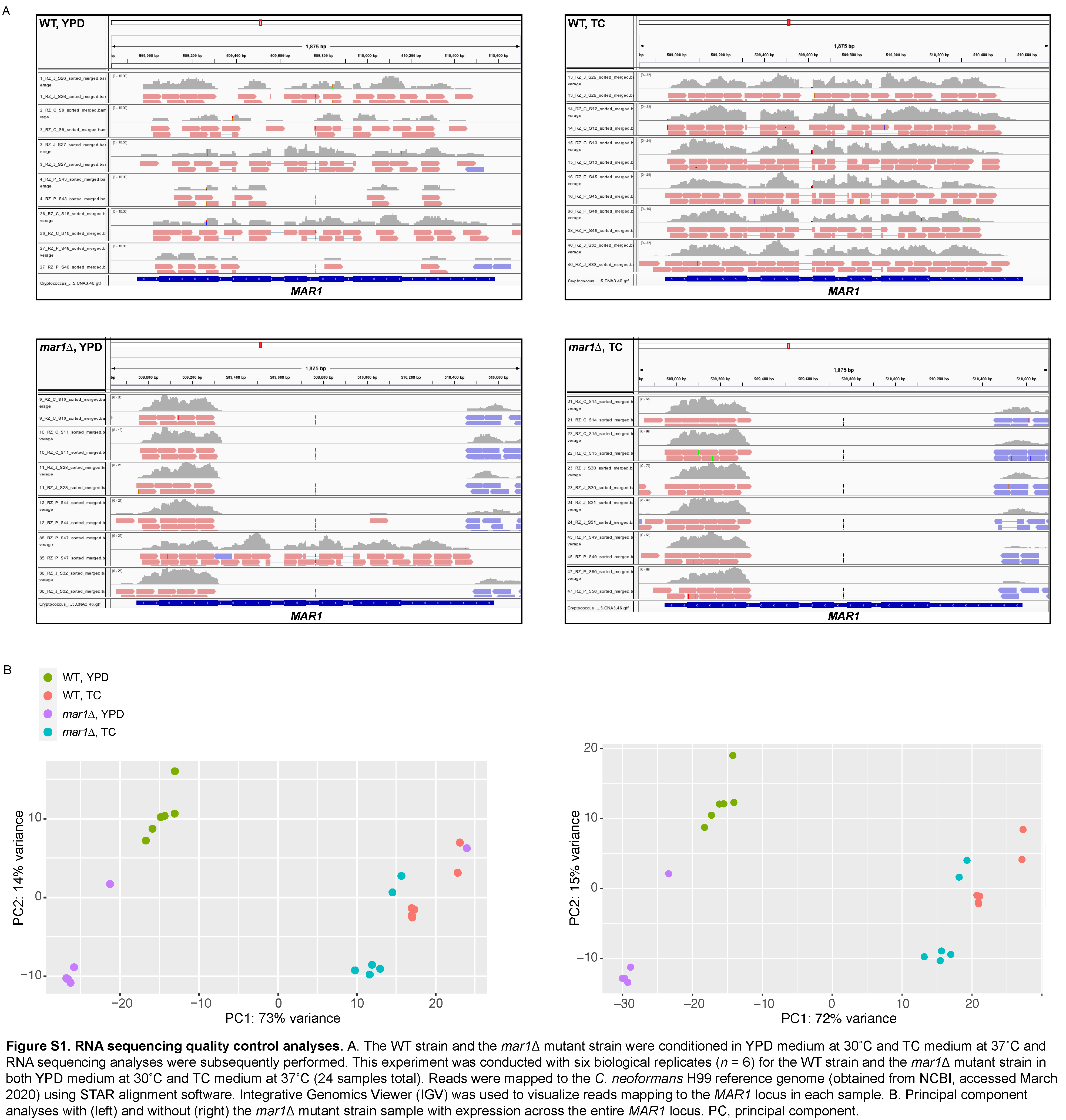

Supplement: Supplementary file 7 [file Image1.TIF]

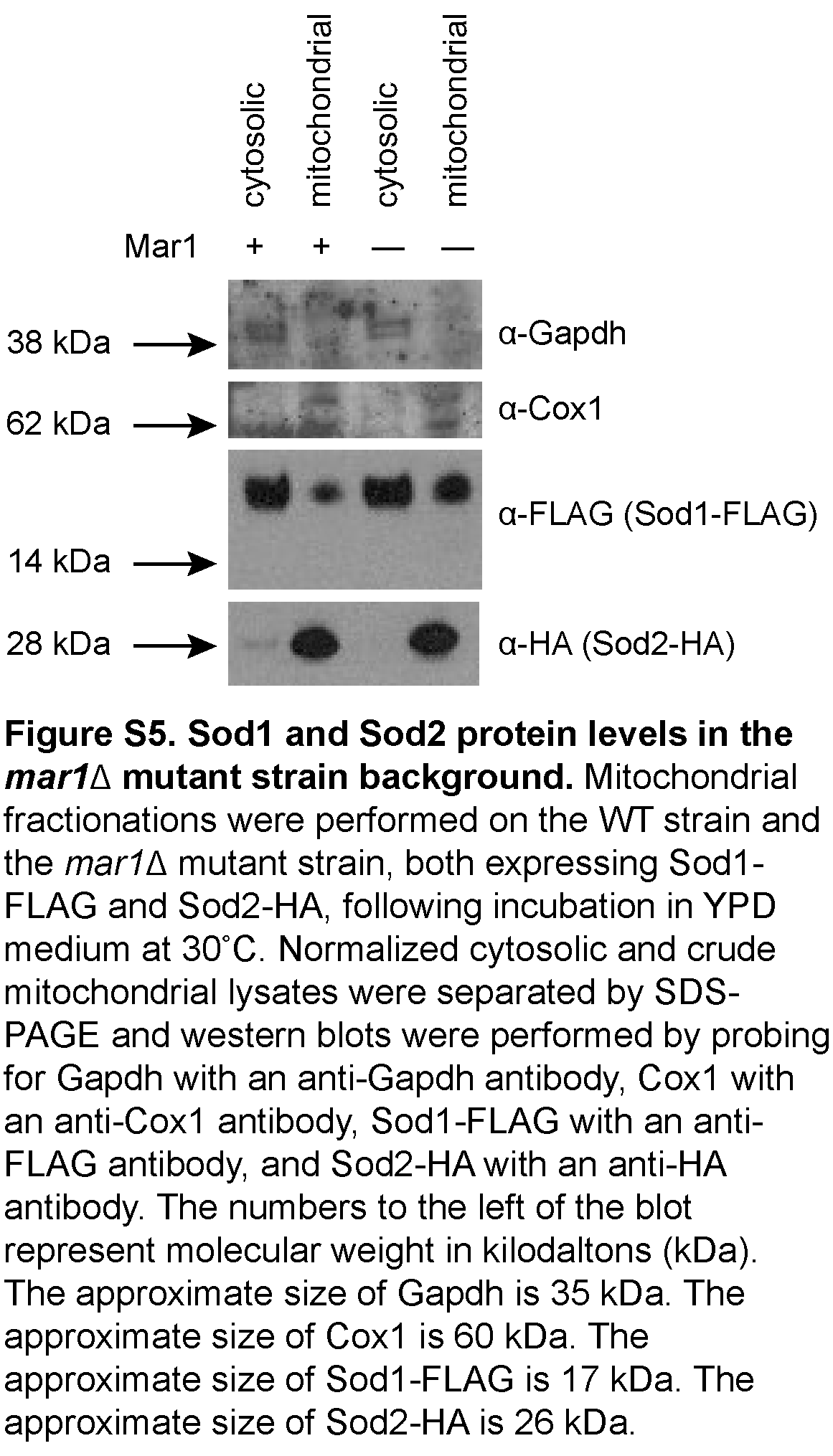

Supplement: Supplementary file 10 [file Image5.TIF]
